# SRI-EEG: State-Based Recurrent Imputation for EEG Artifact Correction

**DOI:** 10.3389/fncom.2022.803384

**Published:** 2022-05-20

**Authors:** Yimeng Liu, Tobias Höllerer, Misha Sra

**Affiliations:** Department of Computer Science, University of California, Santa Barbara, Santa Barbara, CA, United States

**Keywords:** EEG artifact correction, time series imputation, robust EEG sensing, brain computer interface, recurrent neural networks

## Abstract

Electroencephalogram (EEG) signals are often used as an input modality for Brain Computer Interfaces (BCIs). While EEG signals can be beneficial for numerous types of interaction scenarios in the real world, high levels of noise limits their usage to strictly noise-controlled environments such as a research laboratory. Even in a controlled environment, EEG is susceptible to noise, particularly from user motion, making it highly challenging to use EEG, and consequently BCI, as a ubiquitous user interaction modality. In this work, we address the EEG noise/artifact correction problem. Our goal is to detect physiological artifacts in EEG signal and automatically replace the detected artifacts with imputed values to enable robust EEG sensing overall requiring significantly reduced manual effort than is usual. We present a novel EEG state-based imputation model built upon a recurrent neural network, which we call SRI-EEG, and evaluate the proposed method on three publicly available EEG datasets. From quantitative and qualitative comparisons with six conventional and neural network based approaches, we demonstrate that our method achieves comparable performance to the state-of-the-art methods on the EEG artifact correction task.

## 1. Introduction

Electroencephalography (EEG) is a non-invasive and widely-adopted approach to capture surface electrical activity in the human brain with electrodes placed on the scalp. EEG data has been widely used in domains including neuroscience, cognitive science, cognitive psychology, and mental health (Henry, 2006). One line of research has explored EEG as a potential interaction method, e.g., brain computer interfaces (BCIs). Specifically, by extracting context or intention from EEG data, hands-free BCI applications have been tested. Those applications include playing games (van de Laar et al., 2013), controlling a mouse cursor (Aydemir and Kayikcioglu, 2014), controlling robotic arms (Latif et al., 2017), managing smart home appliances (Anindya et al., 2016), and using smartphones (Kumar et al., 2017; Rashid et al., 2018). EEG has also been used as an accessible input modality when traditional input methods are not a feasible option. For example, brain-to-text communication (Willett et al., 2021) and hands-free wheelchair control (Singla et al., 2014) have enabled paralyzed or disabled users to communicate and move without assistance. Moreover, there is a growing number of EEG enabled BCI devices for consumers. Galea^1^, Emotiv^2^, Advanced Brain Monitoring^3^, and Muse^4^ are some representative devices that allow integration of various brain signals into a single headset for use in daily life and virtual reality applications. Research-grade EEG caps such as those developed by OpenBCI^5^ are being used to support both lab-based researchers as well biosensing enthusiasts. In addition, less bulky devices, such as the InEar BioFeed Controller (Matthies, 2013) or the Kokoon EEG-based sleep headphones^6^ are part of a new set of devices called *hearables* designed to track neural and physiological function of a user over long periods of time (Goverdovsky et al., 2017).

Although EEG-aided BCIs have seen significant interest and development since the 1970s, an open and challenging research question is to build robust user experiences under unconstrained (i.e., general and everyday use) conditions, given that EEG signals are easily contaminated by artifacts from users and their environment (Aricò et al., 2018). To deal with artifacts, the recorded EEG signals usually need to be heavily processed by sophisticated software, proprietary algorithms, or manual inspection before use. For example, independent component analysis (ICA) is a widely used approach that plays an important role in the EEG pre-processing phase for artifact removal. Though ICA variants and other advanced methods (e.g., canonical correlation analysis, wavelet transform algorithm, empirical-mode decomposition) have been introduced over the years, the EEG artifact correction procedure still requires manual inspection of the signal data to pick out noise . Furthermore, random and irregularly-occurring artifacts make signal correction increasingly challenging. The complex, time consuming, and labor intensive pre-processing requirements thus limit the robustness and generalization of EEG supported BCIs for many consumer use cases, and hinder the development of ubiquitous EEG-based applications. Therefore, developing an automatic EEG artifact correction approach that can significantly reduce manual tagging time and expense is imperative to making BCI broadly useful as a general purpose input method.

Automated artifact correction methods using deep-learning-based signal processing have achieved state-of-the-art performance on a variety of time series data. One line of research has explored the use of recurrent neural networks (RNNs) for time series data imputation to predict noisy or missing portions from the remaining segments of data. A large set of relevant works [e.g., GRU-D (Che et al., 2018a), MR-HDMM (Che et al., 2018b), MRNN (Yoon et al., 2017), GAIN (Yoon et al., 2018), and BRITS (Cao et al., 2018)] have shown that RNNs can effectively encode the temporal relationships in time series data and are, thus, able to achieve satisfactory performance on the data imputation task. Additionally, transformer-based neural networks have achieved impressive performance in various research domains including natural language processing and computer vision. Motivated by their success, recent work has started to tackle time series data in a sequence-to-sequence manner by applying the self-attention mechanism common in transformer-based models. For example, Zerveas et al. (2021) and Tran et al. (2021) present two representative works to adapt transformer-based encoders on the classification, regression and imputation problems in time series data and achieve superior performance.

While EEG is a type of time series data, the automatic imputation designed for correcting artifacts, caused by EEG's susceptibility to various types of noise during data collection, remains a relatively unexplored and challenging area. To address this challenge and take advantage of recent success of deep learning methods for time series data imputation, we propose a state-based recurrent neural network (RNN) for EEG artifact correction (SRI-EEG) in an automatic manner. Our network is built upon an RNN as its backbone structure. It takes into account the state information encoded in EEG signal representing features of stimuli applied during EEG data recording. Added to the state information is a spatial decay matrix to model EEG channel dependencies. We evaluate the performance of our proposed network on three publicly available datasets: Bike (Bullock et al., 2015), Kaggle (Margaux et al., 2012), and SMR (Tangermann et al., 2012) (Section 6).

In summary, our main contributions include: (1) an automatic imputation method to correct irregularly-occurring artifacts in EEG data leading to significantly reduced manual effort that could encourage broader usage of EEG based interaction modalities; (2) a novel bidirectional long short-term memory (LSTM) model for imputing artifacts in EEG signals. This approach captures the temporal and spatial dependencies of recorded data, and leverages EEG state features to enable automatic artifact correction through data imputation; (3) results from a qualitative and quantitative evaluation of SRI-EEG on three publicly available EEG datasets. Experimental results show that our algorithm achieves state-of-the-art performance on EEG imputation.

## 2. Related Work

### 2.1. Time Series Imputation

Time series data imputation is defined as replacing data gaps with predicted values computed from the remaining data. Simple methods replace the missing data with the mean or median of non-empty values, or the last observed value. Such methods offer a fast and easy way to impute missing portions from known data. However, these approaches are based on simple statistical rules and can easily introduce large errors, e.g., imputing long-range missing segments from limited known data.

Non-deep-learning-based techniques have been commonly adopted as they outperform the aforementioned simple imputation methods and do not demand dense computation. *K* nearest neighbors (KNNs)-based imputation (Zhang and Zhou, 2007) utilizes the statistical dependency in neighboring data, and imputes missing parts using the mean of *K* nearest neighbors. Factorization-based techniques (Friedman et al., 2001), decompose the non-missing signal into basis vectors to approximate values at missing points. Adaptations of factorization-based algorithms, such as multivariate imputation by chained equations (MICE) (Azur et al., 2011) and SoftImpute (Mazumder et al., 2010), have also been explored with varying levels of success. However, one of the main limitations of these approaches is the inability to capture long-term temporal dependencies in time series data.

Recent advancements in deep learning have shown desirable capability to encode long-range temporal correlations and have achieved state-of-the-art imputation performance on time series datasets (Moritz and Bartz-Beielstein, 2017; Luo et al., 2018; Miller et al., 2018; Yuan et al., 2018; Suo et al., 2019; Zhang and Yin, 2019; Tang et al., 2020; Miao et al., 2021; Zerveas et al., 2021). Among these works, BRITS (Cao et al., 2018) and MRNN (Yoon et al., 2017) use bidirectional RNNs, which impute missing values based on hidden states updated in both the forward and backward directions. These two approaches have achieved state-of-the-art performance on one of the most popular multivariate time series datasets, PhysioNet Challenge 2012 (Goldberger et al., 2000). MRNN takes the values to-be-imputed as constraints and does not encode correlations between the missing segments. BRITS does not impose strong assumptions on the imputation setup such as linear dynamics in the hidden states, and enables imputing correlated missing segments.

Motivated by the successful adoption of bidirectional RNNs on the time series imputation problem, we build our network's backbone using a bidirectional LSTM. The LSTM processes time series data in both the forward and backward directions with two separate sequences of hidden states. The two sequences are then averaged to predict imputed values. Such a backbone network has mostly been used for general time series data in prior work (e.g., air quality, human activity, and health care datasets). It fails to impute EEG signals containing a large amount of irregularly occurring artifacts. To tackle this limitation, we introduce encoded EEG state constraints into the network along with taking as input the spatial correlations between signal collected by all the electrodes in an EEG cap. Through our evaluation, reported in Section 6, we demonstrate the effectiveness of our approach targeted at the EEG artifact imputation problem.

### 2.2. EEG Artifact Correction

Artifacts in EEG are undesired noise mainly originating from two types of sources: (1) extrinsic artifacts include environment noise and experimental errors, and (2) intrinsic artifacts include physiological artifacts (Jiang et al., 2019) such as body motions or eye movements. Extrinsic artifacts can be eliminated by filtering recorded signal or following proper experimental procedures. However, preventing intrinsic artifacts is more challenging and their removal requires particular algorithms (Anderer et al., 1999). In this work, we focus on eliminating physiological artifacts stemming from the human body, specifically body motions, eye movements, and cardiac activities. Therefore, in this section we focus on work most closely related to handling physiological artifacts in EEG data.

Blind source separation (BSS) approaches, including principal component analysis (PCA) and independent component analysis (ICA), are widely used for EEG artifact removal. PCA (Berg and Scherg, 1991) is a widely used BSS technique. It decomposes EEG signals into uncorrelated variables, called principal components (PCs), through an orthogonal transformation. The PCs representing artifacts are removed to denoise the signal, while the remaining PCs are used to reconstruct clean EEG data. PCA typically fails to unravel signal dependencies and can mistakenly discard non-artifact signal (Casarotto et al., 2004). ICA (Somers and Bertrand, 2016) is a flexible BSS method. It assumes that collected EEG signals are linear mixtures of artifacts and non-artifacts. The first step involves decomposing recorded signals into independent components (ICs). Sejnowski (1996) introduces artifactual and non-artifactual ICs as independent ICs, such that obvious artifactual ICs can be segregated easily. By eliminating ICs representing artifacts, ICA reconstructs clean signal with the remaining ICs. Built upon the conception of ICA, recent research has studied EEG artifact removal with various ICA variants and demonstrated their effectiveness (Flexer et al., 2005; Bian et al., 2006; Li et al., 2006; Ting et al., 2006; Zhou and Gotman, 2009; Winkler et al., 2011; Rejer and Górski, 2015, 2019; Dimigen, 2020; Klug and Gramann, 2020). Although a large number of ICA variants have been developed over the last twenty years to control EEG artifacts in different scenarios, the inherent idea of how ICA works imposes assumptions on the recorded signal but the assumptions are not always satisfied. For example, ICA can estimate non-Gaussian signals (one of the premises of ICA), but recorded signals are usually unknown to be Gaussian or non-Gaussian (Jiang et al., 2019).

Though ICA is an effective standard for EEG artifact correction in general, using ICA alone still requires careful manual effort and time to inspect the source signal, which may not have satisfactory properties to fit ICA. We use ICA to detect artifacts followed by automatically imputing them using our proposed method. Combining ICA with the proposed automatic imputation approach achieves improved performance compared to using ICA alone as shown in Section 6.1.

### 2.3. Brain Computer Interfaces and Brain Signals

Depending on the features of interest, EEG based BCIs can be categorized into two classes (Lotte, 2015): (1) event-related potential (ERP) based BCI, and (2) oscillatory activity based BCI. ERP BCIs can detect high-amplitude and low-frequency brain responses to known stimuli. They usually contain well-stereotyped waveforms and are robust across subjects (Fazel-Rezai et al., 2012). The robustness of ERPs thus enables efficient learning of ERP features using machine learning based algorithms. For example, the P300 response, one type of ERP, is evoked by visual stimuli, and forms identifiable electrical waveforms. Other types of ERPs, such as motor imagery, also induce recognizable waveforms. Prior deep-learning-based methods have deployed various classifiers to distinguish between different ERP types (Lawhern et al., 2018; Santamaŕıa-Vázquez et al., 2019, 2020; Wen et al., 2019; Zhao et al., 2020; Zang et al., 2021). Oscillatory BCIs can detect the signal power of EEG frequency bands. Due to low signal-to-noise ratio and great variations across subjects (Pfurtscheller and Neuper, 2001), oscillatory BCIs are typically challenging to model using machine learning models. An example of oscillatory BCIs is the sensorimotor rhythm (SMR). Subjects produce lower SMR amplitudes when the corresponding sensorimotor regions are active, and higher amplitudes otherwise (Arroyo et al., 1993). SMR data is greatly variable across and even within subjects, and thus demands subject training for data collection, and longer signal calibration session for practical use (Krusienski et al., 2008).

To assess the generality and robustness of the proposed EEG artifact imputation method under different EEG paradigms, we evaluate SRI-EEG on two ERP datasets: Bike (Bullock et al., 2015) and Kaggle (Margaux et al., 2012), and one oscillatory dataset: SMR (Tangermann et al., 2012).

## 3. Method

**Figure 1:**
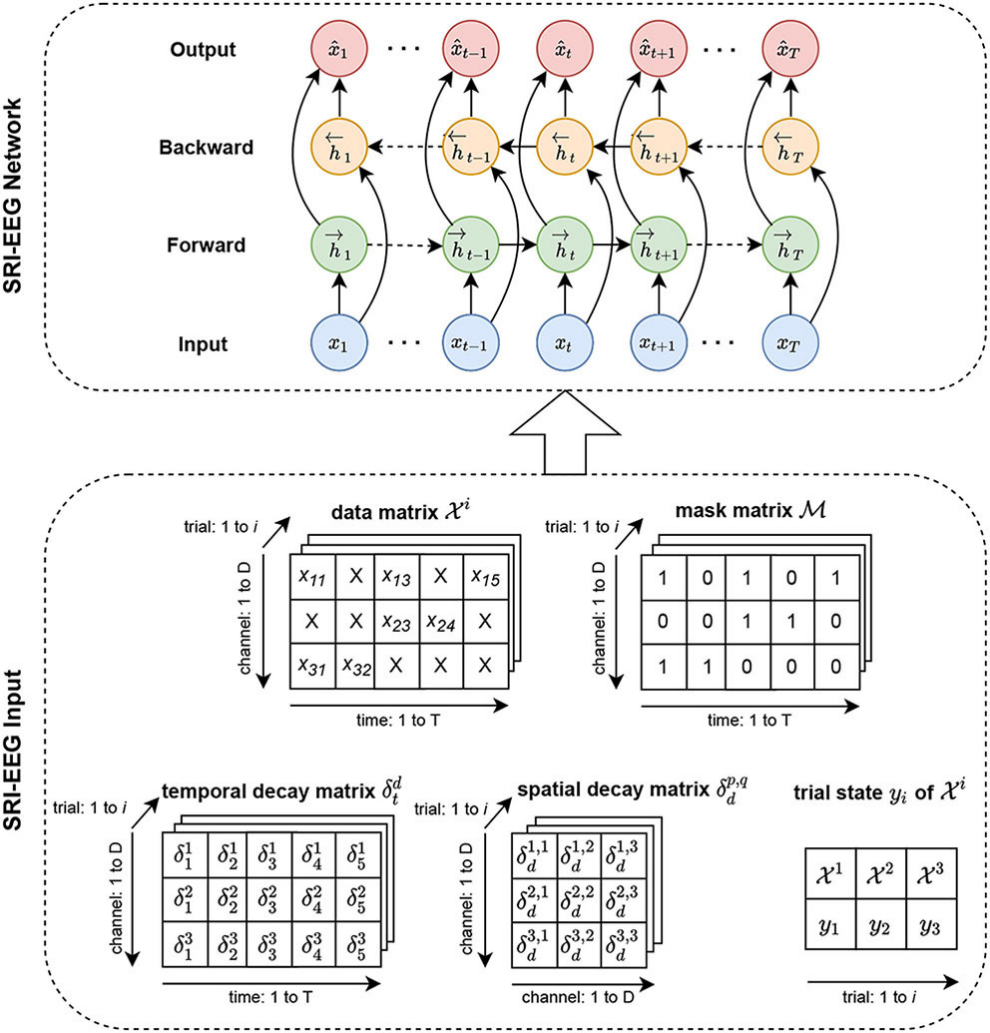
SRI-EEG input and network structure. In the *SRI-EEG Input*, based on the EEG data matrix $X^{i}$, we compute the mask matrix M, and the temporal decay matrix $\delta_{t}^{d}$. Additionally, we generate a spatial decay matrix $\delta_{d}^{p,q}$ based on channel locations in the EEG cap. A trial state matrix *y*_*i*_ is also calculated for each EEG trial. All the matrices computed for a single trial *i* are passed as input to the *SRI-EEG Network*. We adopt a bidirectional LSTM as the backbone network where the hidden states are updated in both the forward and backward directions. Once the imputed values are obtained, mean squared error is computed, and back-propagated for subsequent iterations.

Similar to the standard bidirectional LSTM for time series data imputation, our network takes as input a data matrix, a mask matrix and a time gap matrix processed from raw data. However, unlike using a bidirectional LSTM designed for general time series data, we also take into account a spatial gap matrix for modeling EEG channel dependency. Added to that is a state vector of encoded EEG event features to handle irregularly occurring artifacts that form a typical but challenging problem for EEG based BCI modalities. Section 3.1 introduces the preliminaries covering notation and definitions. Section 3.2 presents the backbone bidirectional LSTM for general time series imputation and discusses its limitations. Sections 3.3 and 3.4 present our design based on the RNN backbone network to specifically tackle the EEG imputation problem. Section 3.5 discusses the learning objectives. Figure 1 shows an overview of the SRI-EEG architecture.

### 3.1. Preliminary

We denote the raw EEG data as a two-dimensional matrix where the first dimension represents time: $X^{i}=\left\{x_{1},x_{2},...,x_{t},...,x_{T}\right\}$ is a sequence of data with *T* timestamp observations collected at trial *i*. The *t*-th observation $x_{t}\in {\mathbb{R}}^{D}$ consists of values from *D* EEG channels $\left\{x_{t}^{1},x_{t}^{2},...,x_{t}^{d},...,x_{t}^{D}\right\}$ at timestamp *t*. Note that the time range 1 to *T* and the channels 1 to *D* in this section are from any single trial of data, where trial *i* is defined as a segment/epoch of interest during EEG data collection.

The time difference between successive valid (non-artifact) observations varies due to artifacts of random lengths. To model the changing time gaps between neighboring valid observations, we define a temporal decay matrix: $\delta_{t}^{d}$ at time *t* and channel *d* similar to Cao et al. (2018):


(1) $$\delta_{t}^{d}=\left\{ \begin{array}{l} \;0\;t=1\\ t-(t-1)+\delta_{t-1}^{d}t>1,\;m_{t-1}^{d}=0\\ \;t-(t-1)t>1,\;m_{t-1}^{d}=1 \end{array}\right.$$

where we denote a binary mask matrix $M=\left\{m_{1}^{d},m_{2}^{d},...,m_{t}^{d},...,m_{T}^{d}\right\}$ along the time domain from channel *d*∈*D* to mask out the artifacts in the data matrices. Specifically,


(2) $$m_{t}^{d}=\;\left\{ \begin{array}{l} 1\;x_{t}^{d}\text{at time }t\text{ and from channel }d\text{ is observed and non-artifact}\\ 0\text{ otherwise} \end{array}\right.$$

Additionally, we denote by *y*_*i*_ the EEG state vector representing the state of the *i*-th trial. It is a learned feature vector using EEGNet (Lawhern et al., 2018), one of the state-of-the-art EEG classifiers. EEGNet allows us to extract a state feature vector to represent stimuli applied to subjects at trial *i* (Section 3.4). The state feature vector is fed into our network as supervised information together with the data matrix, mask matrix, temporal decay matrix, and spatial decay matrix (defined in Section 3.3). Figure 1 depicts all the input matrices.

### 3.2. Bidirectional LSTM for Time Series Imputation

In this section, we briefly introduce unidirectional LSTMs, and our backbone network which consists of a bidirectional LSTM with input mask and temporal decay matrices. Following the background information, we discuss the backbone structure's limitations and introduce our improvements.

#### 3.2.1. Unidirectional LSTM

In a unidirectional LSTM, the hidden layer receives an input vector *x*_*t*_, and predicts an output vector ${\hat{x}}_{t}$. At each timestamp *t*, the hidden layer maintains a hidden state *h*_*t*_, and updates the hidden state based on the current input *x*_*t*_ and the previous hidden state *h*_*t*−1_. We formulate this procedure as:


(3) $$\begin{array}{r} h_{t}=\sigma_{h}\left(W_{xh}x_{t}+W_{hh}h_{t-1}+b_{h}\right) \end{array}$$

where σ_*h*_ is the hidden layer's activation function, *W*_*xh*_ is the weight matrix from the input layer to the hidden layer, *W*_*hh*_ is the weight matrix between two temporally consecutive hidden states, and *b*_*h*_ is the hidden layer's bias vector. Consequently, we compute the output vector ${\hat{x}}_{t}$ as:


(4) $$\begin{array}{r} {\hat{x}}_{t}=\sigma_{x}\left(W_{x}h_{t}+b_{x}\right) \end{array}$$

where σ_*x*_ is the output layer's activation function, *W*_*x*_ is the weight matrix from the hidden layer to the output layer, and *b*_*x*_ is the output layer's bias vector.

#### 3.2.2. Unidirectional LSTM With Input Mask

The unidirectional LSTM takes continuous input without data gaps to update hidden states progressively. However, for the imputation task, where the input data matrix $X^{i}$ consists of both artifacts and non-artifacts, the raw data cannot be fed into the network to update the hidden states. The artifacts will induce distracting features and thus introduce undetermined bias in the imputation process. To resolve this issue, we leverage a mask matrix M to filter out artifacts and pass only the valid data into the network, similar to Cao et al. (2018) and Yoon et al. (2018). Accordingly, the input matrix $X^{i}=\left\{x_{1}^{d},x_{2}^{d},...,x_{t}^{d},...,x_{T}^{d}\right\}$ is computed by:


(5) $$\begin{array}{r} x_{t}^{d}=m_{t}^{d}⊙x_{t}+\left(1-m_{t}^{d}\right)⊙{\hat{x}}_{t} \end{array}$$

where ⊙ is element-wise multiplication.

#### 3.2.3. Unidirectional LSTM With Input Mask and Temporal Decay

Although the hidden states of LSTMs enable the modeling of decayed influence along the time axis (i.e., distant values have less impact than close ones), the length of effective time window is automatically learned and thus not easily controllable by hyper-parameter tuning. To address this limitation, we utilize the defined temporal decay matrix $\delta_{t}^{d}$ to control the temporal influence on hidden state update, similar to Cao et al. (2018). Specifically, the temporal decay factor $\beta_{t}^{d}$ is computed by:


(6) $$\begin{array}{r} \beta_{t}^{d}=\exp\left\{-\max\left(0,W_{\beta }\delta_{t}^{d}+b_{\beta }\right)\right\} \end{array}$$

where *W*_β_ is the time gap's weight matrix, and *b*_β_ is the bias vector. Consequently, the hidden state is updated by applying an element-wise multiplication between the previous hidden state (at timestamp *t*−1) and the current temporal decay factor (at timestamp *t*):


(7) $$\begin{array}{r} h_{t}=\sigma_{h}\left(W_{xh}x_{t}+W_{hh}\left[h_{t-1}⊙\beta_{t}\right]+b_{h}\right) \end{array}$$

where β_*t*_ represents the temporal decay factor at time *t* from all the *D* channels.

#### 3.2.4. Bidirectional LSTM With Input Mask and Temporal Decay

We denote by $\vec{h}$ the hidden state sequence of the forward-directional LSTM, and we iteratively update the sequence based on inputs from timestamps 1 to *T*. Conversely, the backward-direction LSTM updates the hidden state sequence $\overset{⃖}{h}$ by taking in reversed inputs from timestamps *T* to 1. Combining both the forward and backward hidden states, we compute the estimated values ${\hat{x}}_{t}$ by:


(8) $$\begin{array}{r} {\hat{x}}_{t}=\sigma \left(W_{x}^{\text{forward}}\vec{h_{t}}+b_{x}^{\text{forward}},W_{x}^{\text{backward}}\overset{⃖}{h_{t}}+b_{x}^{\text{backward}}\right) \end{array}$$

where σ is an average function to combine the forward and backward hidden states. $W_{x}^{\text{forward}}$ and $b_{x}^{\text{forward}}$ are the forward-directional LSTM's weight matrix and bias vector from the hidden layer to the output layer, respectively. It is similar for $W_{x}^{\text{backward}}$ and $b_{x}^{\text{backward}}$ in the backward-direction LSTM.

#### 3.2.5. Our Improvements

Introducing the mask matrix and temporal decay into the bidirectional LSTM proposed by prior work forms an effective backbone network to impute multiple time series datasets as evaluated by Yoon et al. (2017) and Cao et al. (2018). Nevertheless, EEG is a special type of time series data with unique characteristics such as spatial dependency between electrodes and state-related global waveforms. For example, when a region of the human brain is stimulated by a certain event, the imputation is likely to benefit from assigning higher weights to the EEG signals recorded on the scalp above that active brain region, e.g., all electrodes above the motor cortex. Additionally, the imputation performance can be potentially improved by capturing EEG waveform patterns from EEG sequences in a different trial that are temporally similar, i.e., in the same EEG state. Such EEG state correlation introduces favorable inductive biases. We take into account the aforementioned EEG properties and design the imputation algorithm by exploring the channel/electrode spatial correlations (Section 3.3), including EEG state modeling (Section 3.4).

### 3.3. Learning the Spatial Correlations of EEG Channels

**Figure 2:**
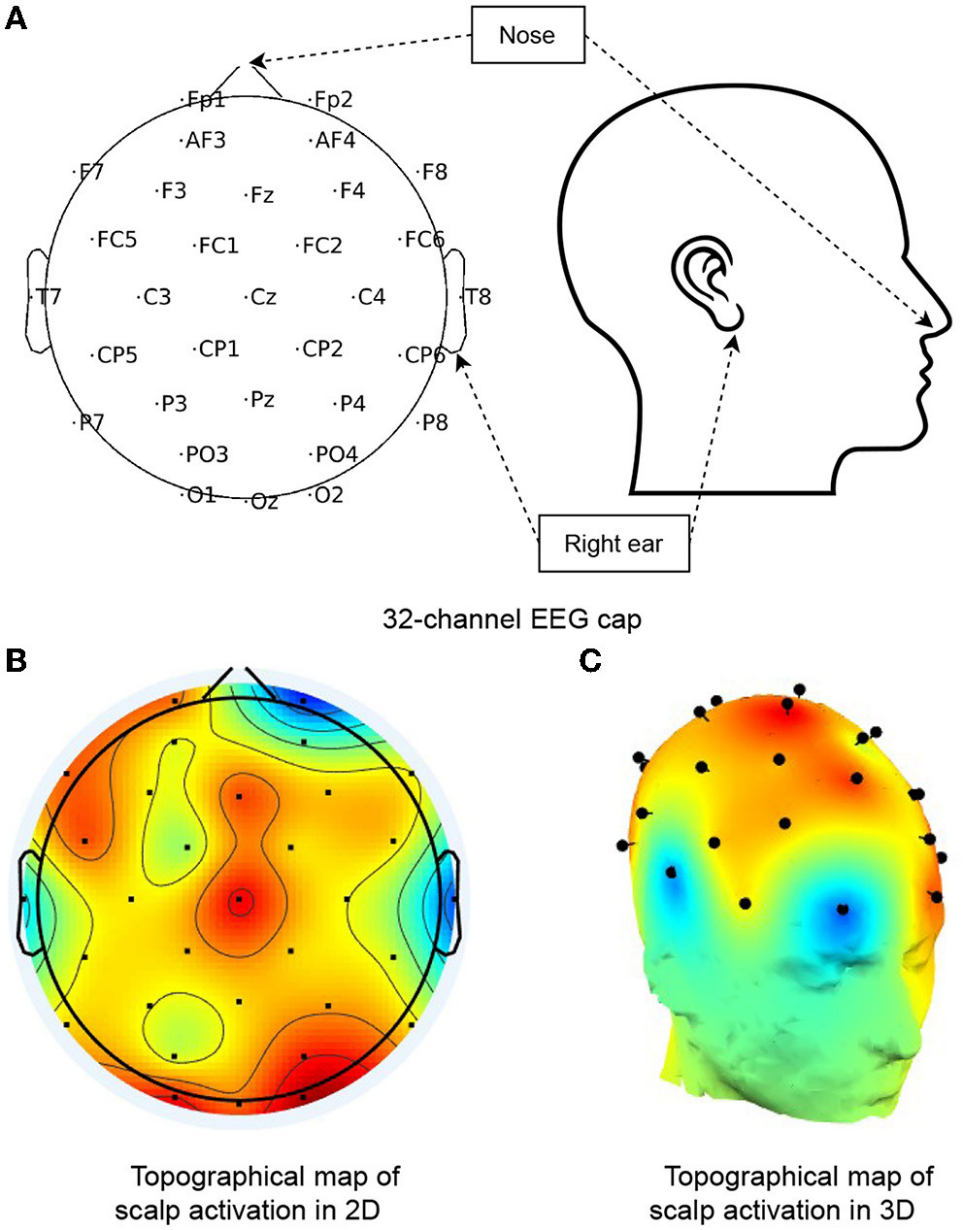
Topological map of a 32-channel EEG cap. **(A)** Shows the channel distribution of a 32-electrode EEG cap (as used in the Bike dataset) with channel names, nose, and right ear annotated. **(B,C)** Present an example of cortex activity in 2D and 3D topographical maps, respectively. The red regions signify active, while blueness stands for the opposite, and the gradual change of colors are computed by linear interpolations between channel signals.

As shown in Figure 2, the 32 electrodes are positioned on a cap in a particular layout (the International 10-20 system). The electrode topology presents varying proximity between different pairs of electrodes. For example, when certain brain regions are active, triggered by some stimuli (red regions in Figure 2), all the electrodes within that region collect related signals. These signals, thus, become important neighboring values to impute contaminated channels affected by artifacts. To model the spatial correlations between the electrodes, which prior work has not extensively studied, we propose a novel spatial decay matrix $\delta_{d}^{p,q}$. It consists of the Euclidean distance between any EEG channel pair: *p* and *q*. We position the channels in a 3D Euclidean coordinate system, and denote the coordinates of any two channels as *p* = (*p*_*x*_, *p*_*y*_, *p*_*z*_) and *q* = (*q*_*x*_, *q*_*y*_, *q*_*z*_), respectively. Accordingly, the distance between any EEG electrode pair is calculated by:


(9) $$\begin{array}{r} \delta_{d}^{p,q}=||p-q|| \end{array}$$

Similar to the temporal decay factor β_*t*_, we introduce a spatial decay factor η_*d*_ to model the decay dependency between EEG channel pairs as their distance increases:


(10) $$\begin{array}{r} \eta_{d}=\exp\left\{-\max\left(0,W_{\eta }\delta_{d}+b_{\eta }\right)\right\} \end{array}$$

where δ_*d*_ denotes the distances between any channel *d* and all the remaining channels, *W*_η_ is the weight matrix, and *b*_η_ is the bias vector.

Combining the temporal and spatial decay factors with the weight hyper-parameter λ∈[0, 1], we formulate the temporal-spatial decay matrix as:


(11) $$\begin{array}{r} \gamma_{t,d}=\beta_{t}+\lambda \eta_{d} \end{array}$$

We update the bidirectional LSTM's hidden states by element-wise multiplying the temporal-spatial decay matrix and the last hidden state (forward and backward):


(12) $$\begin{array}{r} \begin{array}{c} \vec{h_{t}}=\sigma_{h}\left(W_{xh}^{\text{forward}}x_{t}+W_{hh}^{\text{forward}}\left[h_{t-1}⊙\gamma_{t,d}\right]+b_{h}^{\text{forward}}\right)\\ \overset{⃖}{h_{t}}=\sigma_{h}\left(W_{xh}^{\text{backward}}x_{t}+W_{hh}^{\text{backward}}\left[h_{t+1}⊙\gamma_{t,d}\right]+b_{h}^{\text{backward}}\right) \end{array} \end{array}$$

### 3.4. Modeling the State of EEG Trials

Studies on EEG classification (Lotte et al., 2007; Fazel-Rezai et al., 2012; Lawhern et al., 2018) have demonstrated that different stimuli applied to subjects during each trial can lead to different features in the collected EEG data. These features can be differentiated to enable classifying the EEG signals, e.g., classifying ERPs. Motivated by these findings and to take advantage of the recognizable features of EEG waveforms, we utilize the state features to provide supervised information on the EEG signal imputation task. Specifically, we obtain the features learned from the state-of-the-art EEG classifier EEGNet (Lawhern et al., 2018). We subsequently transform the features into a 1-dimensional vector (state vector) to serve as supervised information for our network's input. More formally, we denote the state of EEG trial *i* as *y*_*i*_, and concatenate *y*_*i*_ with the masked data matrix to pass them together into the network.

In summary, the update of hidden states considering temporal-spatial decay and the EEG state is computed by:


(13) $$\begin{array}{r} \begin{array}{c} \vec{h_{t}}=\sigma_{h}\left(W_{xh}^{\text{forward}}\left(x_{t}⊕y_{i}\right)+W_{hh}^{\text{forward}}\left[h_{t-1}⊙\gamma_{t,d}\right]+b_{h}^{\text{forward}}\right)\\ \overset{⃖}{h_{t}}=\sigma_{h}\left(W_{xh}^{\text{backward}}\left(x_{t}⊕y_{i}\right)+W_{hh}^{\text{backward}}\left[h_{t+1}⊙\gamma_{t,d}\right]+b_{h}^{\text{backward}}\right) \end{array} \end{array}$$

where ⊕ signifies concatenation.

### 3.5. Learning Objective

We train our model using the Mean Squared Error (MSE) loss in the back-propagation process:


(14) $$\begin{array}{r} L_{mse}=\frac{1}{n}\cdot \overset{T}{\underset{t=1}{\sum }}\left(1-m_{t}\right){\left(x_{t}-{\hat{x}}_{t}\right)}^{2} \end{array}$$

where *n* denotes the total number of observations in each trial. The MSE is calculated as an averaged sum of the difference between the observed and imputed EEG values along the time domain and across all EEG channels.

## 4. EEG Dataset

For evaluation, we use three publicly available datasets, namely Bike, Kaggle^7^, and SMR^8^. The feature type, the number of subjects and samples, and the number of trials per subject in all three datasets are summarized in Table 1.

**Table 1:** Summary of the EEG datasets used in this study.

**Dataset**	**Feature type**	**No. of subjects**	**No. of samples (K)**	**Trials per subject**
SMR	Oscillatory	9	2.5	288
Kaggle	ERP	26	8.8	340
Bike denoised_1 Hz	ERP	11	66	128
Bike denoised_2 Hz	ERP	8	48	64
Bike noisy_1 Hz	ERP	11	66	512
Bike noisy_2 Hz	ERP	8	48	256

### 4.1. Bike

The Bike dataset was introduced by Bullock et al. (2015) and provides P300 event related potentials (ERPs). Twelve subjects performed two different versions of three-stimuli oddball tasks while seated on a stationary bike. Participants were asked to respond to the target stimuli and ignore the remaining two types of distracting stimuli. The stimuli were presented at different rates: 1 Hz [200 ms stimulus presentation with 800 ms inter-stimulus interval (ISI)], and 2 Hz (200 ms stimulus presentation with 300 ms ISI). Participants completed the two-version tasks at rest (sat on the bike but not pedaling), and during exercise (sat on the bike and pedaling).

We followed the train-test split protocol and pre-processing procedure used in Ding et al. (2019). We used both the 1 and 2 Hz datasets (pedaling or noisy, and not pedaling or denoised). Specifically, the Noisy_1 Hz and Noisy_2 Hz datasets are the “noisy” versions of the Bike dataset since pedaling introduced a large amount of artifacts in the collected EEG signals. Additionally, we analyzed “denoised” versions of the Bike dataset, named Denoised_1 Hz and Denoised_2 Hz, where the subjects were not pedaling during EEG collection. For the data pre-processing, we applied a bandpass filter and kept the 1–40 Hz frequency band and lower the sampling rate from 512 to 128 Hz.

### 4.2. Kaggle

For the Kaggle dataset (Margaux et al., 2012), 26 participants engaged in a P300 speller task. The system presented a random sequence of flashing letters and numbers, arranged in a 6 ×6 grid, to elicit the P300 responses (Krusienski et al., 2008). The goal of this task was to determine whether the displayed item is the target item by analyzing recorded EEG signals. Following the same pre-processing procedure as the Bike dataset, the EEG signal was band-pass filtered to 1–40 Hz and down-sampled to 128 Hz. The training and testing sets were split as suggested in the official release.

### 4.3. SMR

We use the BCI Competition IV Dataset 2A (Tangermann et al., 2012) involving nine subjects for oscillatory EEG data analysis. The SMR data consists of four classes of imagined movements of left and right hands, feet and tongue. The EEG data was originally recorded using 22 electrodes, sampled at 250 Hz and bandpass filtered between 0.5 and 100 Hz (Schirrmeister et al., 2017). We re-sampled the data to 128 Hz, and band-pass filtered it to 1–40 Hz to keep consistent pre-processing setups. The training and testing sets were split as in the official release.

## 5. Experimental Setup

### 5.1. Evaluation Setup and Metrics

We train our imputation model using the Adam optimizer (Kingma and Ba, 2015) with a learning rate of 0.001 and batch size of 32. The weight λ between temporal and spatial decay factors is set to 0.8 which was determined to be a suitable value through experimentation. We adopt the early stopping strategy by randomly selecting 15% of the training sets to form the validation dataset. Training is stopped when the lowest validation error is obtained. The training weights are saved for subsequent evaluation on the testing sets.

We adopt two metrics for performance evaluation: mean absolute error (MAE) and root mean squared error (RMSE). We define $G^{i}=\left\{g_{1},g_{2},...,g_{n}\right\}$ as the observed data matrix, and $P^{i}=\left\{p_{1},p_{2},...,p_{n}\right\}$ as the corresponding imputed data matrix. *n* is the total number of observations in each trial *i*. Accordingly, the metrics are defined as:


(15) $$\begin{array}{r} \text{MAE}=\frac{1}{n}\overset{n}{\underset{k=1}{\sum }}|g_{k}-p_{k}| \end{array}$$

(16) $$\begin{array}{r} \text{RMSE}=\sqrt{\frac{1}{n}\overset{n}{\underset{k=1}{\sum }}{\left(g_{k}-p_{k}\right)}^{2}} \end{array}$$

### 5.2. Baseline and Comparison Methods

We compare the proposed imputation model with the following non-deep-learning algorithms: Mean, KNN, SoftImpute, and ICA, and with two deep learning algorithms: MRNN and BRITS. The non-deep-learning approaches compute comparatively fast and are widely adopted as baselines in prior studies. The deep-learning methods have achieved great improvements in imputation performance on time series datasets allowing us comparisons with the state-of-the-art.

- Mean: Mean imputation replaces artifacts with the global average along the time domain for each trial.
- KNN^9^: KNN uses the *k*-nearest neighbors to find similar samples, and imputes the artifacts with weighted average of the *k* neighbors.
- SoftImpute^9^[]: SoftImpute is a matrix completion based technique that works by applying iterative soft thresholding of SVD decompositions.
- ICA^10^: ICA decomposes the original signal into independent components, and then removes the components consisting of artifacts for denoising the signal. ICA is a popular EEG artifact correction approach widely adopted by researchers.
- MRNN^11^ (Yoon et al., 2017): MRNN is a bidirectional RNN based algorithm, and treats the imputed values as constants without modeling correlations between channels.
- BRITS (see textfootnote 11) (Cao et al., 2018): BRITS is an extension of MRNN. It considers the correlations between different imputed values.

### 5.3. Dataset Preparation

We evaluate our proposed method on two setups: (1) we detect artifacts in EEG with ICA and manual inspection, and impute the detected artifacts using our proposed imputation method, and (2) we synthesize EEG artifacts by removing varying percentages of the recorded EEG data, and impute the synthesized artifacts using our proposed imputation method.

#### 5.3.1. EEG Artifact Detection

We extract when and where the artifacts occur during EEG recording *via* ICA (Winkler et al., 2011). Additionally, we detect obvious missing values caused by bad channels using functions in EEGLAB (Delorme and Makeig, 2004) by manually investigating kurtosis in the channel and looking at how well each channel correlates with the surrounding channels.

Although this setup needs manual effort to detect artifacts in the recorded EEG signal, it lays a practical basis to evaluate our network's effectiveness. This setup also incurs complexity in extracted artifacts, such as different artifact quantities across trials and different artifact patterns caused by various sources of noise.

#### 5.3.2. Synthesized EEG Artifacts

We synthesize EEG noise by randomly removing certain portions of the observed data, and replacing the removed parts with *NaN*s. The synthesized artifact rates in our experiment are: 2.5, 5, 7.5, 10, 12.5, 15, 17.5, 20%. This setup offers controllable artifact rates to compare our approach's performance with other imputation methods at fixed artifact rates. The artifact ratios help to analyze an imputation method's performance on varying artifact rates (studied in Section 6.2).

The motivation for utilizing the synthesized artifacts is to prevent the network from getting scoped by certain types of artifacts, such as those caused by heart beats. This goal is achieved by introducing randomness and increasing complexity of the input data. Specifically, the synthesized artifacts may consist of clean EEG, real artifacts or both. The synthesized random and complex artifacts prevent the model from simply “remembering” artifact patterns but failing to effectively capture the temporal and spatial relationships between data points. By evaluating the imputation methods based on the synthesized artifacts setup, we aim to test the algorithm's robustness and further boost EEG usage for scenarios that introduce different artifact types. The synthesized artifacts are obtained automatically, as opposed to the manual effort in detecting real artifacts using ICA. This setup is therefore, a step toward the potential goal of building automatic EEG artifact correction pipelines where people can detect real artifacts without heavily relying on human effort. Following automatic artifact detection, our proposed imputation algorithm can be applied in the artifact correction process to generate denoised EEG signal.

## 6. Results

### 6.1. Detected Artifacts Imputation

**Figure 3:**
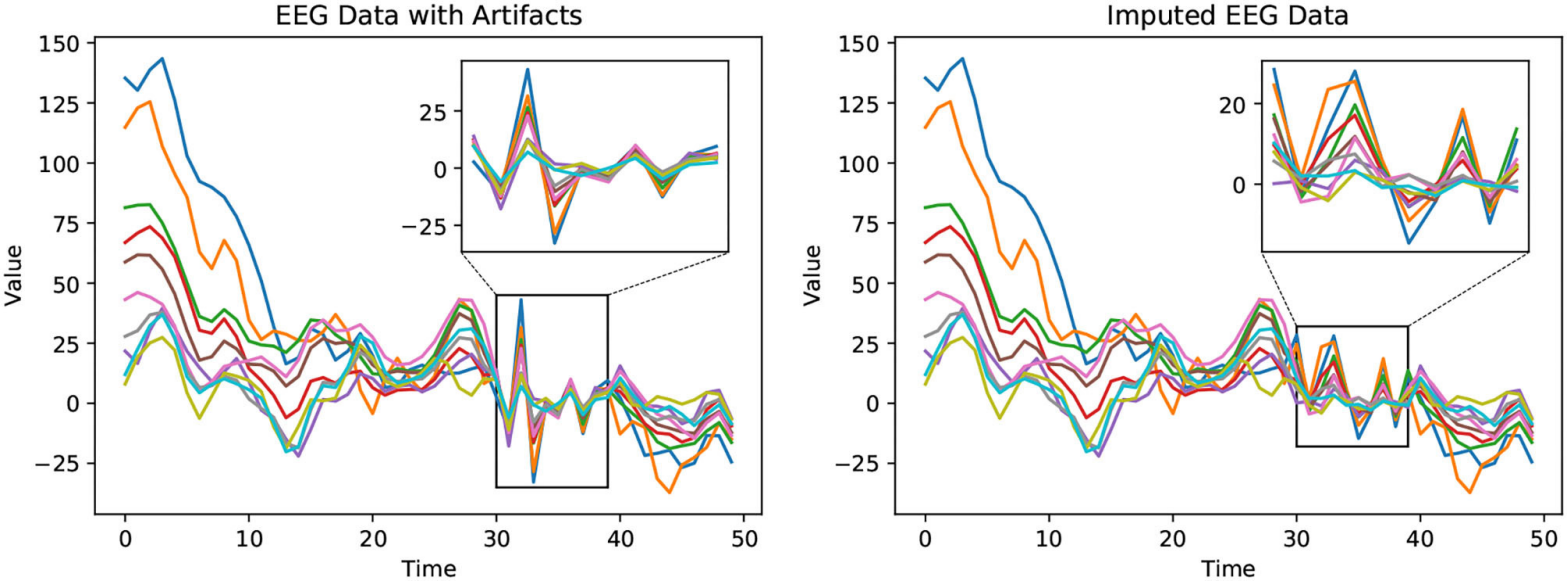
Observed EEG data with artifacts **(left)** and the corrected EEG data with imputed values **(right)**. The plots depict EEG waveforms recorded from ten randomly selected channels (32 channels in total) in the Bike-Denoised_1Hz dataset. In the left figure, values in the rectangle are detected as signals with artifacts. The right figure shows the corrected EEG signal with imputed values in the box. The extreme values caused by artifacts have been corrected by our proposed method, SRI-EEG.

We qualitatively compare the observed EEG data and the corresponding corrected EEG data output by our proposed imputation method, shown in Figure 3. In the Bike-Denoised_1 Hz dataset, we randomly select EEG data collected by 10 out of 32 channels from a random trial. This is done to avoid crowding in the plots while still ensuring generality. Each channel is plotted in a unique color. Marked by the boxes, signals from timestamps 30–40 are detected as contaminated data. The artifacts are visible as mostly extreme values in this time window. After applying our proposed imputation method, as shown in the right plot, the contaminated data has been corrected by taking into account values from neighboring channels and timestamps, and by considering the trial state feature. The imputed values eliminate the waveforms consisting of extreme large or small values to maintain the waveform trend as a whole.

We also conduct a quantitative study and Tables 2, 3 show the detected EEG artifact imputation performance comparisons. The results show great variance across different datasets as the occurrence of detected artifacts differs considerably. The two denoised Bike datasets present lower MAE and RMSE compared with the corresponding noisy sets. For each dataset, we can see an improvement in imputation performance from the top to the bottom. Our method performs well on all three datasets and achieves lower errors compared to the competing approaches in general. Overall, the results demonstrate our proposed method's ability to correct real EEG artifacts.

**Table 2:** Imputation of detected artifacts as evaluated by MAE ± standard deviation.

**Method**	**Denoised_1 Hz**	**Denoised_2 Hz**	**Noisy_1 Hz**	**Noisy_2 Hz**	**Kaggle**	**SMR**
Mean	20.585 ± 0.48	19.219 ± 0.88	80.499 ± 0.46	85.669 ± 0.70	17.070 ± 0.42	1.795 ± 0.24
KNN	17.599 ± 0.27	14.882 ± 0.31	73.415 ± 1.06	81.915 ± 1.02	15.422 ± 0.48	1.045 ± 0.29
SoftImpute	14.610 ± 2.56	13.576 ± 2.54	71.798 ± 3.44	78.643 ± 3.79	14.822 ± 2.89	0.761 ± 0.27
ICA	11.366 ± 1.45	10.675 ± 2.20	66.758 ± 4.70	76.018 ± 5.65	12.611 ± 1.24	0.634 ± 0.18
BRITS	10.603 ± 0.10	10.398 ± 0.13	57.359 ± 1.02	64.854 ± 1.04	**11.363** ±**0.21**	0.634 ± 0.04
MRNN	11.802 ± 0.17	10.156 ± 0.17	48.401 ± 1.13	52.461 ± 1.12	12.545 ± 0.24	0.733 ± 0.07
SRI-EEG	**10.096** ±**0.15**	**8.522** ±**0.10**	**46.048** ±**0.94**	**48.091** ±**0.92**	12.497 ± 0.17	**0.457** ±**0.06**

*Lower is better and the best performance is in bold. Performance averaged over 24 runs on the entire test set*.

**Table 3:** Imputation of detected artifacts as evaluated by RMSE ± standard deviation.

**Method**	**Denoised_1 Hz**	**Denoised_2 Hz**	**Noisy_1 Hz**	**Noisy_2 Hz**	**Kaggle**	**SMR**
Mean	22.936 ± 2.12	21.764 ± 3.76	127.083 ± 3.94	132.164 ± 3.59	77.368 ± 1.94	1.961 ± 0.26
KNN	21.829 ± 2.03	19.829 ± 2.08	119.225 ± 2.65	127.483 ± 3.12	73.746 ± 0.62	1.138 ± 0.35
SoftImpute	20.004 ± 1.85	18.643 ± 2.06	116.865 ± 2.15	122.271 ± 2.36	73.112 ± 1.75	0.896 ± 0.23
ICA	20.082 ± 1.91	15.261 ± 2.94	115.796 ± 2.76	119.766 ± 2.28	70.542 ± 1.49	0.805 ± 0.17
BRITS	19.786 ± 0.30	13.294 ± 0.32	117.366 ± 1.07	118.078 ± 1.07	**68.309** ±**0.22**	0.738 ± 0.05
MRNN	17.943 ± 0.33	13.142 ± 0.37	**97.741** ±**1.12**	95.164 ± 1.12	69.238 ± 0.22	0.827 ± 0.07
SRI-EEG	**17.145** ±**0.24**	**12.204** ±**0.38**	97.803 ± 0.92	**94.281** ±**0.92**	69.053 ± 0.17	**0.724** ±**0.09**

*Lower is better and the best performance is in bold. Performance averaged over 24 runs on the entire test set*.

To measure imputation performance significance on data reported in Table 2, we performed a Wilcoxon signed rank test over all the imputed values averaged over 10 runs on the Denoised-1 Hz, Denoised-2 Hz, Noisy-1 Hz, Noisy-2Hz, Kaggle and SMR test sets. We report the full results in Supplementary Tables 1–6 and Supplementary Figures 1–6. Here we summarize the main findings.

**Denoised_1 Hz dataset**. Absolute error for the imputed values was significantly different for the compared imputation methods as determined using the Friedman test, $X_{\left(6\right)}^{2}=440.15$, *p* < 0.0001, *n* = 1, 980. All pairwise differences between SRI-EEG and the other methods were significant to at least a level of *p* < 0.001. Exception is SRI-EEG vs ICA, which was significant to a level of *p* < 0.05 instead.

**Denoised_2 Hz dataset**. Absolute error for the imputed values was significantly different for the compared methods as determined using the Friedman test, $X_{\left(6\right)}^{2}=369.44$, *p* < 0.0001, *n* = 1, 980. All pairwise differences between SRI-EEG and the other methods were significant to at least a level of *p* < 0.001.

**Noisy_1 Hz dataset**. Absolute error for the imputed values was significantly different for the compared imputation methods as determined using the Friedman test, $X_{\left(6\right)}^{2}=656.41$, *p* < 0.0001, *n* = 1, 980. All pairwise differences between SRI-EEG and the other methods were significant to at least a level of *p* < 0.001. Exceptions are SRI-EEG vs MRNN and SRI-EEG vs BRITS, both of which were non-significant (*p* >0.05).

**Noisy_2 Hz dataset**. Absolute error for the imputed values was significantly different for the compared imputation methods as determined using the Friedman test, $X_{\left(6\right)}^{2}=349.25$, *p* < 0.0001, *n* = 1, 980. All pairwise differences between SRI-EEG and the other methods were significant to at least a level of *p* < 0.001.

**Kaggle dataset**. Absolute error for the imputed values was significantly different for the compared imputation methods as determined using the Friedman test, $X_{\left(6\right)}^{2}=2317.41$, *p* < 0.0001, *n* = 1, 980.

**SMR dataset**. Absolute error for the imputed values was significantly different for the different compared methods as determined using the Friedman test, $X_{\left(6\right)}^{2}=1967.3$, *p* < 0.0001, *n* = 1, 980. All pairwise differences between SRI-EEG and the other methods were significant to at least a level of *p* < 0.001.

### 6.2. Synthesized Artifacts Imputation

#### 6.2.1. Imputation With the Same Synthesized Artifact Rate

Tables 4, 5 show the performance of different imputation methods evaluated on all the datasets with 10% artifact rate. Evidently, simply replacing artifacts with the mean of observed data is highly inaccurate. KNN and SoftImpute perform much better than simple averaging imputation. Using ICA to detect and remove artifacts outperforms the aforementioned three methods. In contrast, the two RNN based methods, BRITS and MRNN, demonstrate significantly improved performance compared to the non-deep-learning-based approaches. SRI-EEG shows comparable performance to BRITS and MRNN.

**Table 4:** Imputation of 10% synthesized artifacts as evaluated by MAE ± standard deviation.

**Method**	**Denoised_1 Hz**	**Denoised_2 Hz**	**Noisy_1 Hz**	**Noisy_2 Hz**	**Kaggle**	**SMR**
Mean	22.198 ± 0.31	20.710 ± 0.66	75.448 ± 0.51	83.967 ± 0.58	18.638 ± 0.43	1.284 ± 0.14
KNN	17.305 ± 0.26	14.559 ± 0.48	71.093 ± 0.92	79.626 ± 0.96	16.886 ± 0.37	1.039 ± 0.21
SoftImpute	13.604 ± 1.54	13.026 ± 1.78	69.850 ± 1.82	77.839 ± 1.97	15.601 ± 1.19	0.838 ± 0.17
ICA	12.287 ± 0.79	11.495 ± 1.47	62.581 ± 1.20	75.032 ± 1.71	14.595 ± 1.39	0.796 ± 0.16
BRITS	11.859 ± 0.39	8.419 ± 0.39	56.348 ± 0.81	61.161 ± 0.95	12.940 ± 0.11	0.712 ± 0.04
MRNN	10.648 ± 0.33	8.244 ± 0.28	46.677 ± 0.75	50.870 ± 1.48	12.636 ± 0.10	0.724 ± 0.02
SRI-EEG	**9.964** ±**0.42**	**7.796** ±**0.37**	**44.739** ±**0.68**	**43.184** ±**1.76**	**12.501** ±**0.10**	**0.698** ±**0.01**

*Lower is better and the best performance is in bold. Performance averaged over 24 runs on the entire test set*.

**Table 5:** Imputation of 10% synthesized artifacts as evaluated by RMSE ± standard deviation.

**Method**	**Denoised_1 Hz**	**Denoised_2 Hz**	**Noisy_1 Hz**	**Noisy_2 Hz**	**Kaggle**	**SMR**
Mean	24.673 ± 3.72	22.447 ± 3.20	120.794 ± 3.86	131.776 ± 3.83	77.093 ± 1.68	1.386 ± 0.17
KNN	21.774 ± 1.62	19.335 ± 1.75	114.307 ± 1.93	124.389 ± 1.98	75.064 ± 0.52	1.274 ± 0.23
SoftImpute	20.791 ± 1.71	18.424 ± 1.87	112.380 ± 2.69	121.201 ± 2.85	74.314 ± 1.34	1.051 ± 0.16
ICA	20.482 ± 0.98	15.948 ± 1.62	113.583 ± 1.72	118.057 ± 2.15	72.794 ± 1.47	0.983 ± 0.20
BRITS	20.399 ± 0.77	13.474 ± 0.51	115.515 ± 2.08	116.477 ± 2.12	70.321 ± 1.13	0.911 ± 0.04
MRNN	18.539 ± 0.65	13.021 ± 0.62	96.492 ± 1.49	93.212 ± 1.86	72.594 ± 0.72	0.925 ± 0.03
SRI-EEG	**17.267** ±**0.71**	**12.142** ±**0.45**	**91.074** ±**1.71**	**88.192** ±**1.70**	**69.985** ±**0.85**	**0.889** ±**0.01**

*Lower is better and the best performance is in bold. Performance averaged over 24 runs on the entire test set*.

#### 6.2.2. Imputation With Varying Synthesized Artifact Rates

**Figure 4:**
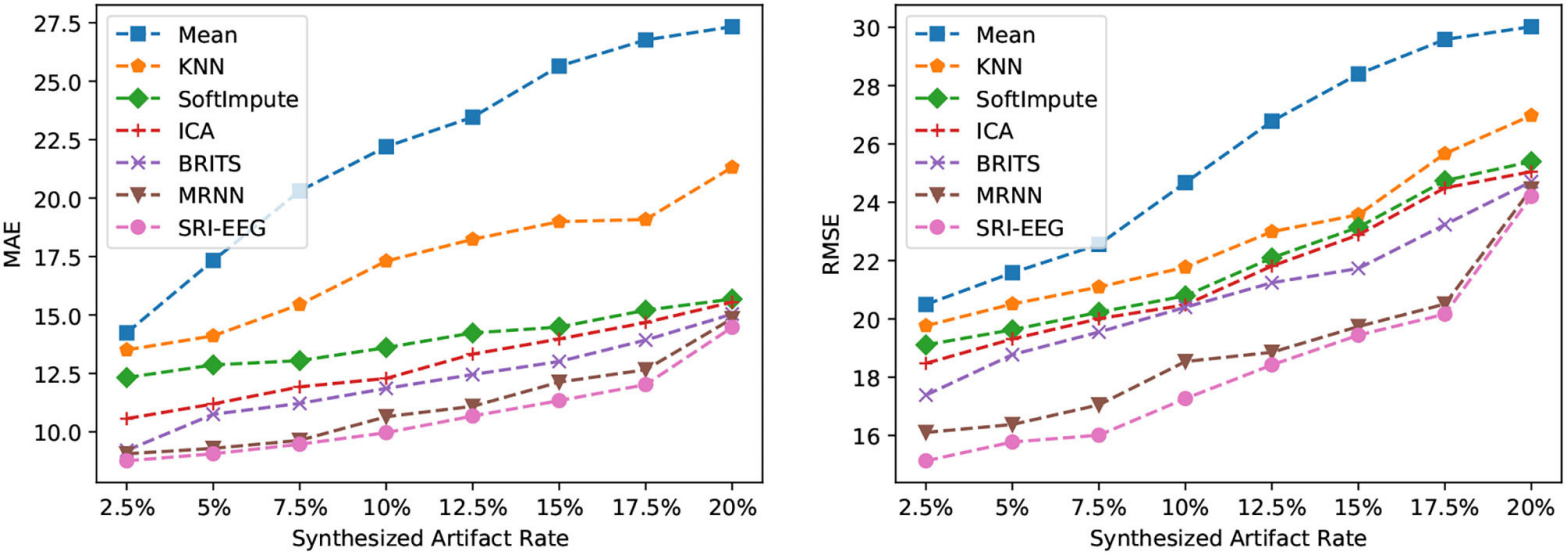
Imputation performance of the different synthesized artifact rates on the Bike-Denoised_1Hz dataset. **(Left)** Performance evaluated by MAE. **(Right)** Performance evaluated by RMSE. For both metrics, lower is better.

On the Bike-Denoised_1Hz dataset, we evaluate the imputation performance at various artifact rates. Figure 4 reveals the MAE (left) and RMSE (right) using different imputation methods at artifact rates from 2.5 to 20%. As is clear, the two errors increase with increase in artifact rates. In particular, simple mean imputation is very sensitive to artifacts, where the errors grow rapidly as the number of artifacts increases. KNN, SoftImpute, and ICA perform much better than the mean imputation. The two RNN based methods (BRITS and MRNN) and our method achieve further lower imputation errors than the non-RNN based approaches at all artifact rates. This study demonstrates the robustness of our model's ability to handle varying proportions of artifacts in the signal, and the effectiveness of deep-learning-based methods in general to tackle the imputation problem at different artifact rates.

### 6.3. Ablation Study

#### 6.3.1. Influence of EEG Channel Spatial Dependency and EEG Trial State

We perform an ablation study to evaluate the effectiveness of including EEG state and channel spatial correlation as the network input. As shown in Tables 6, 7, after removing either or both the trial state supervision and spatial decay in SRI-EEG, the imputation performance degenerates on all the datasets evaluated by MAE and RMSE. Based on these results, both factors appear to be important components contributing to our algorithm's performance demonstrating effectiveness for the EEG imputation task compared to other methods.

**Table 6:** Ablation study of EEG state and channel spatial correlation in SRI-EEG on the three datasets with 10% artifact rate (MAE, lower is better and the best performance is in bold).

**Method**	**Denoised_1 Hz**	**Denoised_2 Hz**	**Noisy_1 Hz**	**Noisy_2 Hz**	**Kaggle**	**SMR**
SRI-EEG	**9.964**	**7.796**	**44.739**	**43.184**	**12.501**	**0.698**
SRI-EEG w/o State	10.120	8.586	47.646	49.790	12.808	0.758
SRI-EEG w/o Space	10.440	9.922	46.389	51.174	12.997	0.762
SRI-EEG w/o StateSpace	12.433	12.972	53.243	57.736	13.105	0.778

**Table 7:** Ablation study of EEG state and channel spatial correlation in SRI-EEG on the three datasets with 10% artifact rate (RMSE, lower is better and the best performance is in bold).

**Method**	**Denoised_1 Hz**	**Denoised_2 Hz**	**Noisy_1 Hz**	**Noisy_2Hz**	**Kaggle**	**SMR**
SRI-EEG	**17.267**	**12.142**	**91.074**	**88.192**	**69.985**	**0.889**
SRI-EEG w/o State	18.717	13.280	96.787	91.841	70.917	0.943
SRI-EEG w/o Space	18.708	13.809	95.391	93.618	71.538	0.924
SRI-EEG w/o StateSpace	20.612	14.958	98.447	94.863	72.687	0.942

### 6.4. Post-imputation Analysis

#### 6.4.1. Goal

To evaluate SRI-EEG on a practical task, we conducted post-imputation analysis in the form of an EEG classification problem. Our aim was to analyze if our model can correct artifacts in EEG and further boost the EEG classification performance. We designed this study as an indirect reflection of the imputation performance and a first step toward using imputation based EEG artifact correction to support EEG-based applications.

#### 6.4.2. Dataset and Metrics

We performed analysis on the Bike dataset, i.e., Denoised_1 Hz, Denoised_2 Hz, Noisy_1 Hz, and Noisy_2 Hz. The Bike dataset consists of P300 ERPs from three-stimuli oddball tasks (introduced in Section 4.1). Therefore, we conduct the classification experiments to evaluate the three-class classification performance.

We use precision, recall and F1 score as the classification metrics:


(17) $$\begin{array}{r} \text{Precision}=\frac{TP}{TP+FP}\\ \text{Recall}=\frac{TP}{TP+FN}\\ \text{F1 score}=2\times \frac{\text{Precision}\times \text{Recall}}{\text{Precision}+\text{Recall}} \end{array}$$

where *TP*, *FP*, and *FN* stand for “true positive,” “false positive,” and “false negative,” respectively.

#### 6.4.3. Setup

We take the classification performance without imputation as a baseline. Specifically, based on the recorded EEG data, i.e., no imputation applied, we perform baseline classification using xDAWN+RG, which is a multi-class EEG classifier (Barachant et al., 2011). Compared against this baseline, we classify the imputed EEG data using BRITS, MRNN, and SRI-EEG as the imputation methods, respectively.

#### 6.4.4. Results and Analysis

We present the classification comparisons in Table 8 with the best performance in bold. Higher precision and recall correspond to better performance. We find that by classifying imputed EEG signals, both the precision and recall are improved in general on the denoised and noisy Bike sets compared with directly classifying the raw data without artifact correction. Importantly, compared with the two RNN-based imputation methods, SRI-EEG achieves improved precision and recall on the classification task evaluated on Denoised_1Hz and Denoised_2 Hz datasets. Although the precision on Noisy_1 Hz and Noisy_2 Hz datasets is less satisfactory than that using MRNN based post-imputation analysis, the recall shows improved performance.

**Table 8:** EEG classification performance comparison.

**Method**	**Denoised_1 Hz**			**Denoised_2 Hz**			**Noisy_1 Hz**			**Noisy_2 Hz**		
	**P**	**R**	**F1**	**P**	**R**	**F1**	**P**	**R**	**F1**	**P**	**R**	**F1**
No Imputation + xDAWN+RG	52.32	70.52	60.07	49.33	67.59	57.03	54.40	74.16	62.76	46.93	64.70	54.40
BRITS + xDAWN+RG	52.36	71.28	60.37	50.92	68.76	58.51	55.50	75.91	64.12	39.16	64.89	48.84
MRNN + xDAWN+RG	52.24	71.31	60.30	51.32	68.78	58.78	**56.44**	76.93	**65.11**	**47.95**	66.57	**55.75**
SRI-EEG + xDAWN+RG	**52.76**	**71.34**	**60.66**	**51.72**	**69.34**	**59.25**	56.17	**77.03**	64.97	39.46	**67.61**	49.83

*Precision %, Recall %, and F1 score % are reported. (Higher is better and the best performance is in bold)*.

To measure any significance in performance based on data in Table 8, we performed a McNemar's test over all the classified labels (1,200 labels) on the Denoised-1 Hz, Denoised-2 Hz, Noisy-1 Hz, and Noisy-2 Hz test sets. McNemar's test has been proven to be a suitable and effective way to compare machine learning classifiers (Dietterich, 1998). We report the full results in Supplementary Tables 7–10. Here we summarize the main findings.

(1) On the Denoised_1Hz dataset, all pairwise differences between imputation with SRI-EEG and other methods were significant to at least a level of *p* < 0.01. Exception is SRI-EEG vs MRNN, which was significant to a level of *p* < 0.05 instead. (2) On the Denoised_2 Hz dataset, all pairwise differences between imputation with SRI-EEG and other methods were significant to at least a level of *p* < 0.05. (3) On the Noisy_1Hz dataset, all pairwise differences between imputation with SRI-EEG and other methods were significant to at least a level of *p* < 0.01. (4) On the Noisy_2 Hz dataset, all pairwise differences between imputation with SRI-EEG and other methods were significant to at least a level of *p* < 0.01. Exception is SRI-EEG vs MRNN, which was significant to a level of *p* < 0.05 instead.

In summary, this study demonstrates that applying imputation for artifact correction can enable using EEG for practical scenarios such as classification. Our proposed EEG imputation technique can effectively recover EEG signals through data imputation and help further improve the post-imputation classification performance.

## 7. Limitations and Future Work

From the assessments presented in Section 6.1, our method achieves improved performance on imputing detected artifacts and thus demonstrates potential for use in real EEG artifact correction. However, in some scenarios when subjects perform long-term body motions during EEG data collection, the artifacts caused by muscle movements and cardiac activities can exist in a long time window or for the entire trial. These long-range artifacts significantly limit the availability of enough clean data which is required for RNN based imputation approaches. We can observe this issue from the experiments on synthesized artifacts (Section 6.2.2): when the artifact rate reaches 17.5% and beyond, the imputation error increases rapidly. Thus, RNN based imputation methods rely on having enough valid signal to predict/impute artifacts confidently. In future work, we plan to tackle this limitation inherent to RNNs by exploring the use of reconstruction based denoising approaches that can aid RNN imputation.

We model the spatial dependency of EEG signals as 3D distances between pairs of electrodes. This setup has been proven effective to handle the linearly decayed influence on the EEG cap across channels. This linear decay dependency holds under the assumption that a single event/state is applied to the subject at each EEG trial and the applied stimulus activates a specific region of human brain. When multiple tasks are taken into account, e.g., click the mouse when you see a certain letter on the screen, multiple cortical regions (e.g., the motor cortex and the visual cortex) are activated. In such cases, a simple distance based decay model may fail to capture the complex spatial relationships between electrodes placed above those cortical regions. Future research should address this limitation by using EEG state to support the modeling of EEG channel relationships knowing that channel state and active brain regions are dependent. New work could also explore dynamic capture and computation of electrode relevance given different stimuli.

## 8. Conclusion

In this paper, we introduced an imputation supported artifact removal algorithm to correct irregular and non-uniformly distributed artifacts that ubiquitously exist in EEG signal data. Our imputation approach enables automatic EEG artifact correction with significantly reduced human effort compared to other methods. Our method is based on a bidirectional LSTM as the backbone network, which has been proven effective by prior work, to learn the temporal dependencies in EEG signal. Into this backbone, we introduce EEG trial state data and spatial correlations between EEG electrodes as supervised information to update the bidirectional hidden states. We evaluated our proposed method *via* qualitative and quantitative analyses. From the evaluations, we demonstrated that SRI-EEG can greatly improve EEG imputation performance under multiple setups on all the tested datasets. Furthermore, we examined our network design with ablation studies and conducted post-imputation analysis to discuss potential applications that can be supported by imputation based EEG artifact correction. We believe that imputation approaches can improve the effectiveness, robustness and generalization of EEG enabled BCI applications, and see our approach as an important step toward that goal.

## Data Availability Statement

The original contributions presented in the study are included in the article/Supplementary Material, further inquiries can be directed to the corresponding author/s.

## Author Contributions

The ideas presented in the paper were formulated through a series of meetings to which all authors contributed. All authors contributed to the conception and design of the evaluation. YL organized the database, performed the experiments and analyses, and wrote the first draft of the manuscript. All authors contributed to manuscript revision, read, and approved the submitted version.

## Funding

This work was supported in part by NSF award IIS-1845587 as well as ONR awards N00014-19-1-2553 and N00174-19-1-0024.

## Conflict of Interest

The authors declare that the research was conducted in the absence of any commercial or financial relationships that could be construed as a potential conflict of interest.

## Publisher's Note

All claims expressed in this article are solely those of the authors and do not necessarily represent those of their affiliated organizations, or those of the publisher, the editors and the reviewers. Any product that may be evaluated in this article, or claim that may be made by its manufacturer, is not guaranteed or endorsed by the publisher.

## Footnotes

^1^Galea: https://openbci.com/community/introducing-galea-bci-hmd-biosensing/.

^2^Emotiv: https://www.emotiv.com/.

^3^Advanced Brain Monitoring: https://www.advancedbrainmonitoring.com/.

^4^Muse: https://choosemuse.com/.

^5^OpenBCI: https://openbci.com/.

^6^Kokoon: https://kokoon.io/.

^7^ https://www.kaggle.com/c/inria-bci-challenge

^8^ http://bbci.de/competition/iv/

^9^KNN and SoftImpute are implemented using the Fancy Impute package: https://github.com/iskandr/fancyimpute.

^10^ICA is implemented using the Predictive Imputer package: https://github.com/log0ymxm/predictive_imputer, and sklearn FastICA: https://scikit-learn.org/stable/modules/generated/sklearn.decomposition.FastICA.html.

^11^MRNN and BRITS are implemented based on the public BRITS repository: https://github.com/caow13/BRITS.
