# Supplementary material for: SRI-EEG: State-Based Recurrent Imputation for EEG Artifact Correction
Source: Front Comput Neurosci. 2022 May 20;16:803384. doi: 10.3389/fncom.2022.803384 (PMC9163298; doi:10.3389/fncom.2022.803384)
Supplement: Supplementary file 1 [file Data_Sheet_1.PDF]

Supplementary Material

1 SIGNIFICANCE ANALYSIS: WILCOXON SIGNED-RANK TEST

This section presents the Wilcoxon signed rank test results for Table 2 in the paper. Wilcoxon signed rank test analysis was performed on six datasets: 1) Denoised_1Hz, 2) Denoised_2Hz, 3) Noisy_1Hz, 4) Noisy_2Hz, 5) Kaggle, and 6) SMR. The corresponding test results are reported in pairs of a result table and comparison chart each: Table S1 and Figure S1 to Table S6 and Figure S6, respectively. For the test results of each dataset, the table details pair-wise method comparisons. Accompanying the table is a figure depicting 95% confidence intervals and pair-wise significance findings.

From our analyses, we see that SRI-EEG achieves statistical significance over all the compared methods on four datasets. One exception is on the Noisy_1Hz dataset, SRI-EEG does not convey significance over BRITS ($p = 0.248$) and MRNN ($p = 1.0$). Our interpretation on the non-significance is: since this dataset is contaminated heavily by artifacts, our method and all the compared methods show great imputation absolute errors as reported in Table 1 of our paper. The great amount of artifacts limits the imputation ability of all the studied methods as imputation counts on the quality and quantity of non-artifact data. Given the limited non-artifact data, different methods tends to make similar mistakes at imputed values. The similarity of erroneous imputation locations across different methods contributes to statistical non-significance. We can see in Table S3, the non-significance not only exists for comparisons involving SRI-EEG, but also commonly shows up between BRITS and MRNN ($p = 1.0$), ICA and BRITS ($p = 0.664$), KNN and SOFTIMPUTE ($p = 1.0$), MEAN and SOFTIMPUTE ($p = 1.0$), and MEAN and KNN ($p = 1.0$). The second exception is on the Kaggle dataset, BRITS achieves the lowest mean absolute error (MAE), 11.363; while SRI-EEG and MRNN achieve comparable performance, MAE = 12.497 and MAE = 12.545 respectively.

With the analyzed limitations, our method still shows statistical significance on all the remaining datasets. The Wilcoxon signed rank test supports SRI-EEG's efficiency on EEG artifact imputation as shown in our paper.

	Group1	Group2	Estimate	Conf.low	Conf.high	p	p.adj	p.adj.signif
1	SRIEEG	MEAN	-5.92	-6.63	-5.22	3.84e-62	8.06e-61	****
2	SRIEEG	KNN	-3.90	-4.52	-3.29	3.4 e-35	7.14e-34	****
3	SRIEEG	SOFTIMPUTE	-2.80	-3.36	-2.25	1.52e-24	3.19e-23	****
4	SRIEEG	ICA	-0.865	-1.40	-0.340	1 e- 3	2.6 e- 2	*
5	SRIEEG	BRITS	-0.530	-0.665	-0.395	2.02e-14	4.24e-13	****
6	SRIEEG	MRNN	-1.28	-1.52	-1.05	2.57e-26	5.4 e-25	****
7	MEAN	KNN	2.22	1.74	2.71	7.55e-20	1.59e-18	****
8	MEAN	SOFTIMPUTE	2.21	1.47	2.97	2.97e- 9	6.24e- 8	****
9	MEAN	ICA	4.85	4.20	5.52	2.55e-51	5.36e-50	****
10	MEAN	BRITS	5.54	4.85	6.25	2.54e-56	5.33e-55	****
11	MEAN	MRNN	4.82	4.11	5.55	3.02e-41	6.34e-40	****
12	KNN	SOFTIMPUTE	0.610	-0.0450	1.28	6.9 e- 2	1 e+ 0	ns
13	KNN	ICA	2.89	2.29	3.49	1.51e-22	3.17e-21	****
14	KNN	BRITS	3.59	2.98	4.22	7.12e-30	1.5 e-28	****
15	KNN	MRNN	2.80	2.18	3.42	1.93e-18	4.05e-17	****
16	SOFTIMPUTE	ICA	2.31	1.67	2.95	1.11e-12	2.33e-11	****
17	SOFTIMPUTE	BRITS	2.33	1.78	2.90	8.32e-18	1.75e-16	****
18	SOFTIMPUTE	MRNN	1.56	1.00	2.14	2.06e- 8	4.33e- 7	****
19	ICA	BRITS	0.595	0.0700	1.13	2.7 e- 2	5.59e- 1	ns
20	ICA	MRNN	-0.0900	-0.650	0.465	7.46e- 1	1 e+ 0	ns
21	BRITS	MRNN	-0.895	-1.09	-0.700	6.84e-20	1.44e-18	****

Table S1. Wilcoxon signed-rank test on Denoised_1Hz dataset. Performance averaged over 10 runs on the Denoised_1Hz test set. Group1 and Group2 columns list the pair-wise compared methods. Comparisons involving SRI-EEG are highlighted in yellow. The Estimate column lists the median over the pair-wise differences between 10-run-average absolute errors at each imputed value using the Group1 and Group2 methods. Conf.low and Conf.high are the confidence interval for the Estimate. P-values are in the column p. The adjusted p-values using Bonferroni correction are shown in the column p.adj. The significance level of adjusted p-values are in the column p.adj.signif. (ns: $p > 0.05$, *: $p < 0.05$, **: $p < 0.01$, ***: $p < 0.001$, ****: $p < 0.0001$)

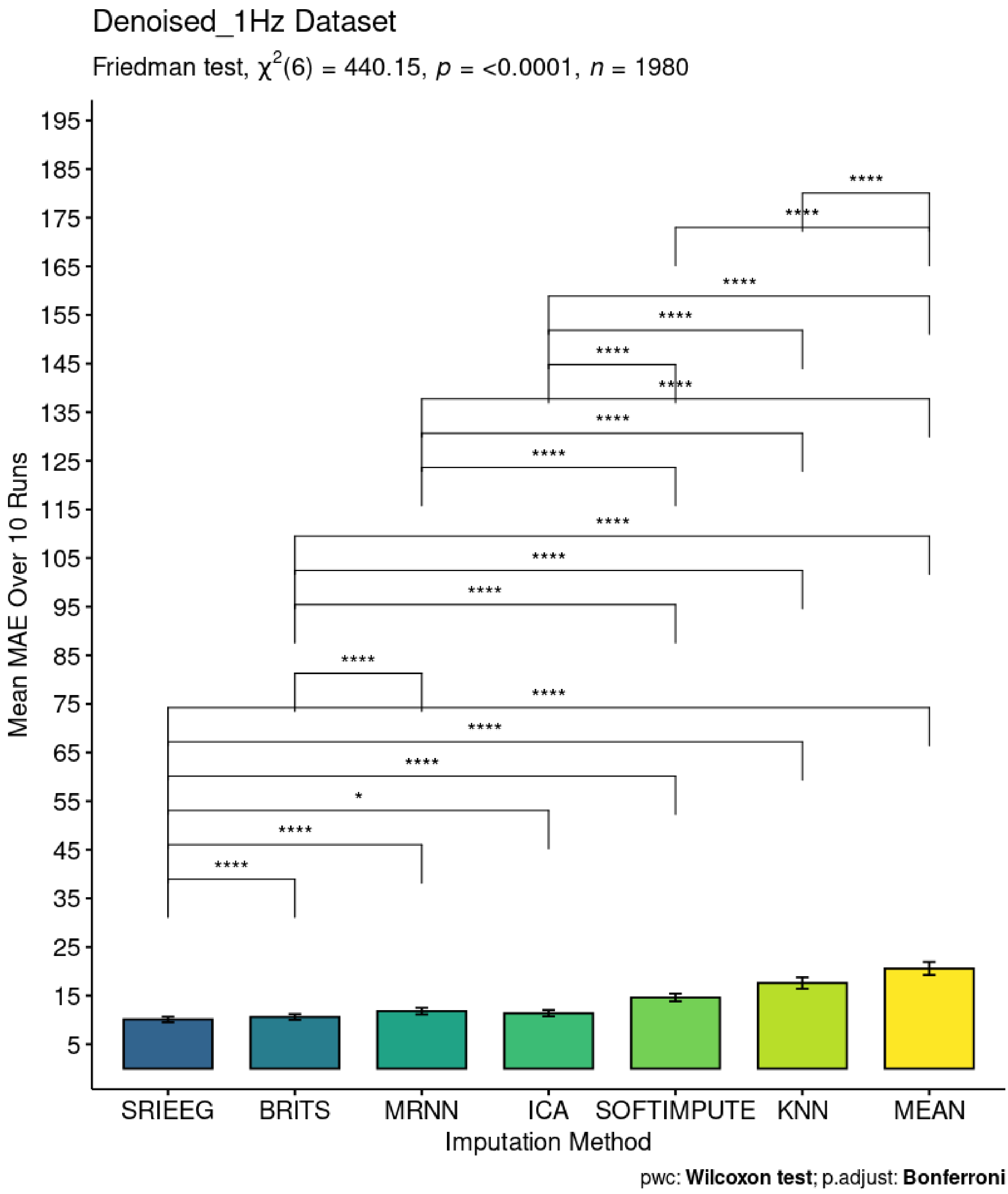

Figure S1. Plot of Denoised_1Hz dataset means over 10 repetitions of mean absolute errors over all imputed values for each imputation method. Error bars show 95% confidence intervals. Significance brackets show results of pair-wise Bonferroni-corrected Wilcoxon signed-rank test.

	Group1	Group2	Estimate	Conf.low	Conf.high	p	p.adj	p.adj.signif
1	SRIEEG	MEAN	-6.45	-7.15	-5.74	2.6 e-76	5.46e-75	****
2	SRIEEG	KNN	-0.975	-1.45	-0.505	4.56e- 5	9.58e- 4	***
3	SRIEEG	SOFTIMPUTE	-3.27	-3.86	-2.70	2.22e-31	4.66e-30	****
4	SRIEEG	ICA	-1.25	-1.72	-0.785	1.41e- 7	2.96e- 6	****
5	SRIEEG	BRITS	-0.605	-0.705	-0.510	4.92e-40	1.03e-38	****
6	SRIEEG	MRNN	-0.0950	-0.120	-0.0650	1.64e-12	3.44e-11	****
7	MEAN	KNN	4.17	3.51	4.85	1.58e-34	3.32e-33	****
8	MEAN	SOFTIMPUTE	2.17	1.44	2.91	2.58e- 9	5.42e- 8	****
9	MEAN	ICA	4.69	4.05	5.34	1.57e-49	3.3 e-48	****
10	MEAN	BRITS	4.64	3.91	5.39	6.74e-37	1.42e-35	****
11	MEAN	MRNN	4.89	4.15	5.64	7.51e-41	1.58e-39	****
12	KNN	SOFTIMPUTE	-2.04	-2.65	-1.44	1.54e-11	3.23e-10	****
13	KNN	ICA	0.410	-0.0800	0.895	1.01e- 1	1 e+ 0	ns
14	KNN	BRITS	-0.560	-1.08	-0.0400	3.5 e- 2	7.39e- 1	ns
15	KNN	MRNN	-0.405	-0.925	0.120	1.27e- 1	1 e+ 0	ns
16	SOFTIMPUTE	ICA	2.23	1.61	2.85	1.48e-12	3.11e-11	****
17	SOFTIMPUTE	BRITS	1.78	1.18	2.39	4.81e- 9	1.01e- 7	****
18	SOFTIMPUTE	MRNN	1.97	1.37	2.58	6.34e-11	1.33e- 9	****
19	ICA	BRITS	-0.430	-0.915	0.0650	8.8 e- 2	1 e+ 0	ns
20	ICA	MRNN	-0.245	-0.735	0.240	3.2 e- 1	1 e+ 0	ns
21	BRITS	MRNN	0.215	0.150	0.285	3.79e-10	7.96e- 9	****

Table S2. Wilcoxon signed-rank test on Denoised_2Hz dataset. Performance averaged over 10 runs on the Denoised_2Hz test set. Group1 and Group2 columns list the pair-wise compared methods. Comparisons involving SRI-EEG are highlighted in yellow. The Estimate column lists the median over the pair-wise differences between 10-run-average absolute errors at each imputed value using the Group1 and Group2 methods. Conf.low and Conf.high are the confidence interval for the Estimate. P-values are in the column p. The adjusted p-values using Bonferroni correction are shown in the column p.adj. The significance level of adjusted p-values are in the column p.adj.signif. (ns: $p > 0.05$, *: $p < 0.05$, **: $p < 0.01$, ***: $p < 0.001$, ****: $p < 0.0001$)

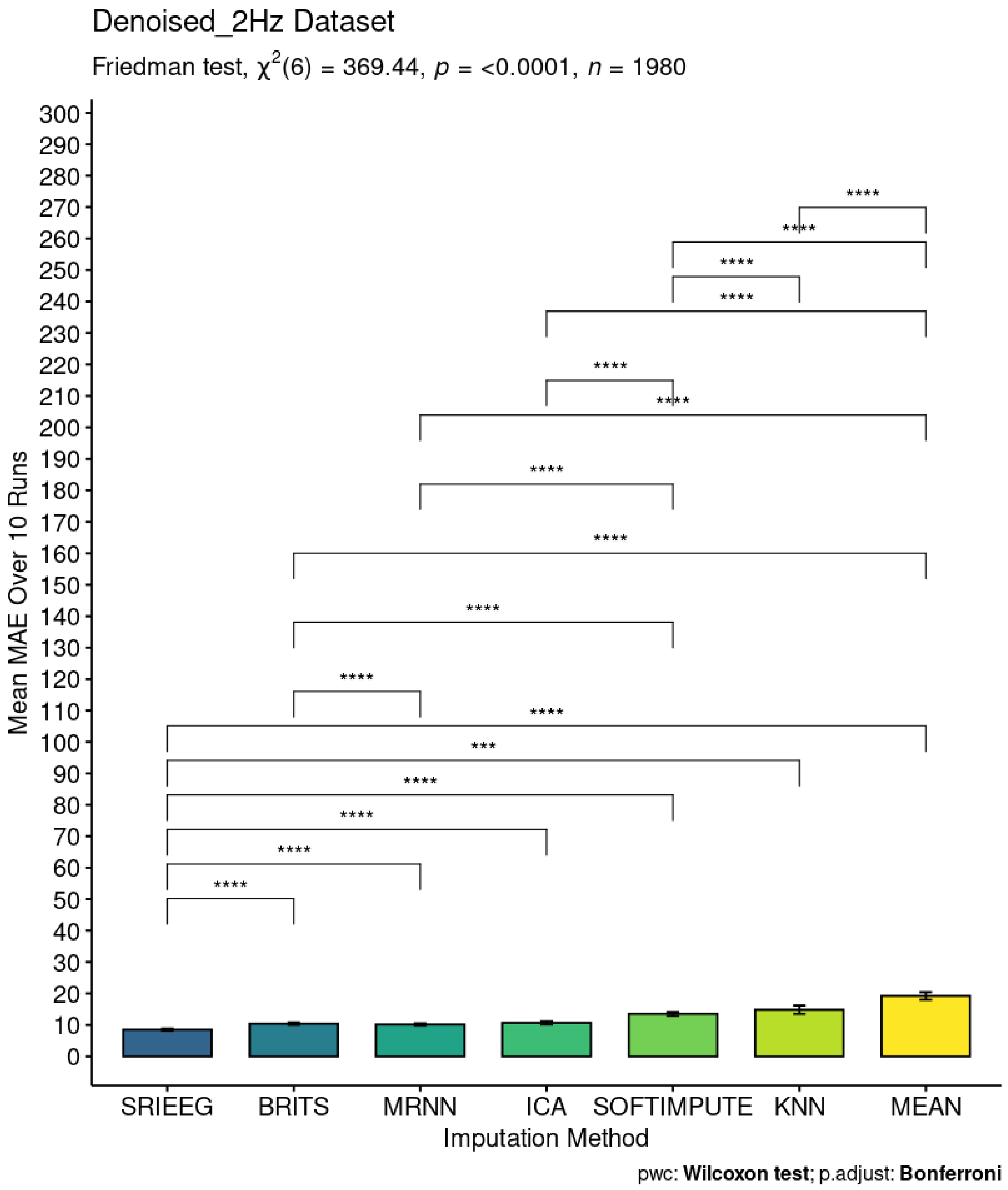

Figure S2. Plot of Denoised_2Hz dataset means over 10 repetitions of mean absolute errors over all imputed values for each imputation method. Error bars show 95% confidence intervals. Significance brackets show results of pair-wise Bonferroni-corrected Wilcoxon signed-rank test.

	Group1	Group2	Estimate	Conf.low	Conf.high	p	p.adj	p.adj.signif
1	SRIEEG	MEAN	-28.1	-31.9	-24.4	4.45e-51	9.35e-50	****
2	SRIEEG	KNN	-29.5	-32.5	-26.5	3.73e-72	7.83e-71	****
3	SRIEEG	SOFTIMPUTE	-26.2	-29.3	-23.1	1.21e-58	2.54e-57	****
4	SRIEEG	ICA	-10.0	-14.7	-5.96	1.11e- 7	2.33e- 6	****
5	SRIEEG	BRITS	-3.62	-6.51	-0.801	1.2 e- 2	2.48e- 1	ns
6	SRIEEG	MRNN	1.44	-0.938	3.77	2.33e- 1	1 e+ 0	ns
7	MEAN	KNN	-0.0550	-3.89	3.80	9.76e- 1	1 e+ 0	ns
8	MEAN	SOFTIMPUTE	2.31	-1.35	6.04	2.17e- 1	1 e+ 0	ns
9	MEAN	ICA	14.6	11.7	17.4	1.12e-22	2.35e-21	****
10	MEAN	BRITS	23.3	19.8	26.7	6.89e-41	1.45e-39	****
11	MEAN	MRNN	25.6	21.9	29.5	9.19e-45	1.93e-43	****
12	KNN	SOFTIMPUTE	2.10	-0.485	4.71	1.11e- 1	1 e+ 0	ns
13	KNN	ICA	12.8	8.88	16.6	4.03e-10	8.46e- 9	****
14	KNN	BRITS	20.8	17.6	23.9	1.03e-33	2.16e-32	****
15	KNN	MRNN	27.0	24.0	30.0	2.91e-65	6.11e-64	****
16	SOFTIMPUTE	ICA	9.38	5.38	13.3	6.41e- 6	1.35e- 4	***
17	SOFTIMPUTE	BRITS	19.7	16.7	22.6	1.49e-34	3.13e-33	****
18	SOFTIMPUTE	MRNN	24.1	21.0	27.1	1.33e-51	2.79e-50	****
19	ICA	BRITS	3.79	0.330	7.31	3.2 e- 2	6.64e- 1	ns
20	ICA	MRNN	8.14	4.33	12.4	6.69e- 6	1.4 e- 4	***
21	BRITS	MRNN	2.49	-0.725	5.69	1.29e- 1	1 e+ 0	ns

Table S3. Wilcoxon signed-rank test on Noisy_1Hz dataset. Performance averaged over 10 runs on the Noisy_1Hz test set. Group1 and Group2 columns list the pair-wise compared methods. Comparisons involving SRI-EEG are highlighted in yellow. The Estimate column lists the median over the pair-wise differences between 10-run-average absolute errors at each imputed value using the Group1 and Group2 methods. Conf.low and Conf.high are the confidence interval for the Estimate. P-values are in the column p. The adjusted p-values using Bonferroni correction are shown in the column p.adj. The significance level of adjusted p-values are in the column p.adj.signif. (ns: $p > 0.05$, *: $p < 0.05$, **: $p < 0.01$, ***: $p < 0.001$, ****: $p < 0.0001$)

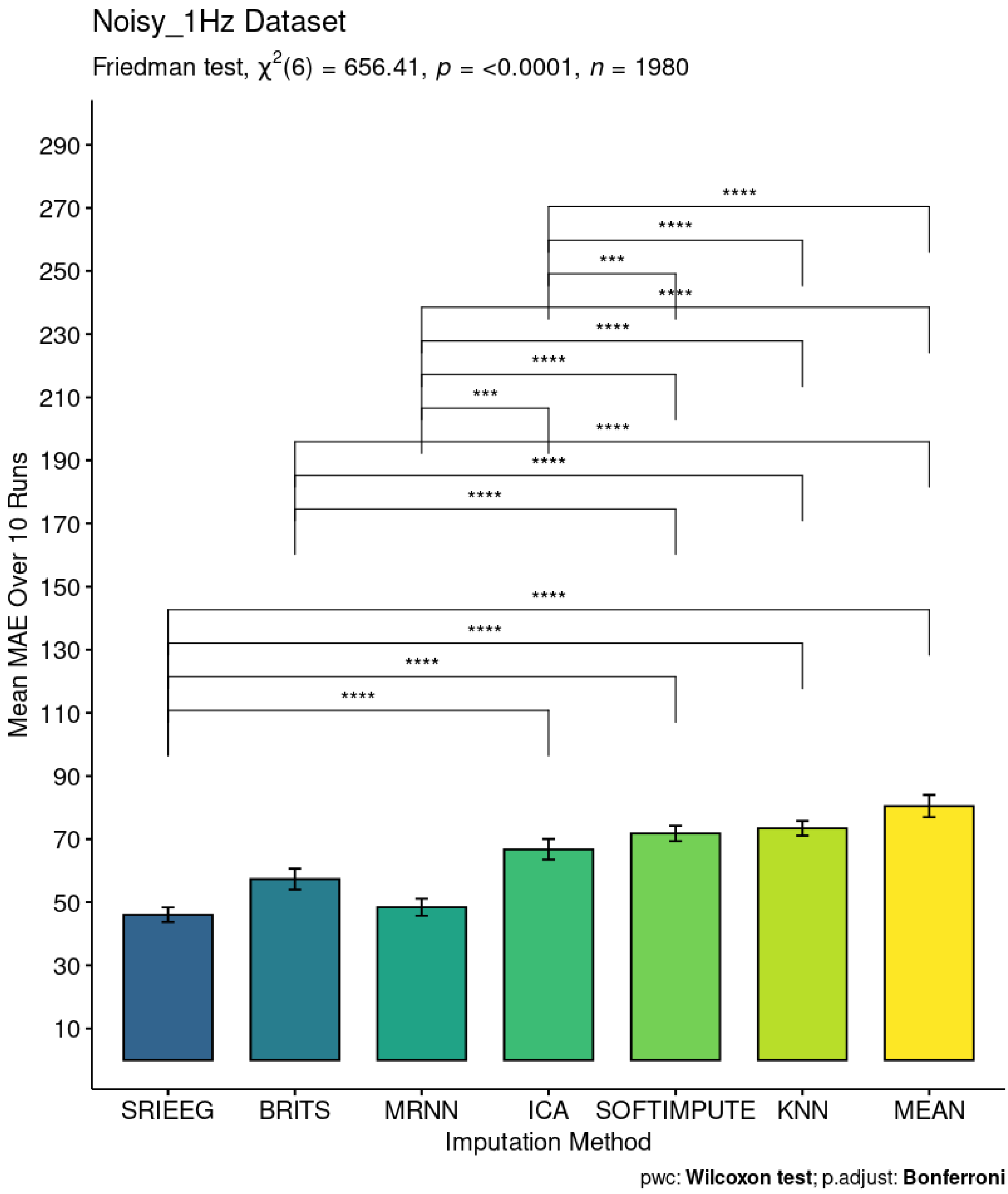

Figure S3. Plot of Noisy_1Hz dataset means over 10 repetitions of mean absolute errors over all imputed values for each imputation method. Error bars show 95% confidence intervals. Significance brackets show results of pair-wise Bonferroni-corrected Wilcoxon signed-rank test.

	Group1	Group2	Estimate	Conf.low	Conf.high	p	p.adj	p.adj.signif
1	SRIEEG	MEAN	-25.2	-28.7	-21.7	5.42e-50	1.14e-48	****
2	SRIEEG	KNN	-28.4	-31.8	-25.0	3.98e-61	8.36e-60	****
3	SRIEEG	SOFTIMPUTE	-24.4	-27.6	-21.3	3.48e-51	7.31e-50	****
4	SRIEEG	ICA	-18.8	-22.3	-15.5	1.75e-30	3.67e-29	****
5	SRIEEG	BRITS	-14.9	-17.6	-12.2	5.8 e-28	1.22e-26	****
6	SRIEEG	MRNN	-5.83	-8.34	-3.33	5.01e- 6	1.05e- 4	***
7	MEAN	KNN	-2.42	-6.47	1.73	2.48e- 1	1 e+ 0	ns
8	MEAN	SOFTIMPUTE	2.96	-0.0650	6.04	5.5 e- 2	1 e+ 0	ns
9	MEAN	ICA	8.47	4.56	12.4	2.6 e- 5	5.46e- 4	***
10	MEAN	BRITS	13.7	10.2	17.3	1.34e-14	2.81e-13	****
11	MEAN	MRNN	21.8	18.1	25.6	1.97e-32	4.14e-31	****
12	KNN	SOFTIMPUTE	4.03	0.420	7.64	2.8 e- 2	5.96e- 1	ns
13	KNN	ICA	3.99	0.325	7.67	3.2 e- 2	6.82e- 1	ns
14	KNN	BRITS	14.1	10.3	18.0	7.58e-13	1.59e-11	****
15	KNN	MRNN	22.5	19.0	26.1	1.25e-37	2.62e-36	****
16	SOFTIMPUTE	ICA	5.38	1.63	9.12	5 e- 3	1.07e- 1	ns
17	SOFTIMPUTE	BRITS	10.9	7.48	14.3	6.83e-10	1.43e- 8	****
18	SOFTIMPUTE	MRNN	19.1	16.1	22.2	1.25e-35	2.62e-34	****
19	ICA	BRITS	3.75	0.330	7.27	3.2 e- 2	6.7 e- 1	ns
20	ICA	MRNN	12.4	8.97	16.0	7.56e-13	1.59e-11	****
21	BRITS	MRNN	9.87	7.06	12.7	7.18e-12	1.51e-10	****

Table S4. Wilcoxon signed-rank test on Noisy_2Hz dataset. Performance averaged over 10 runs on the Noisy_2Hz test set. Group1 and Group2 columns list the pair-wise compared methods. Comparisons involving SRI-EEG are highlighted in yellow. The Estimate column lists the median over the pair-wise differences between 10-run-average absolute errors at each imputed value using the Group1 and Group2 methods. Conf.low and Conf.high are the confidence interval for the Estimate. P-values are in the column p. The adjusted p-values using Bonferroni correction are shown in the column p.adj. The significance level of adjusted p-values are in the column p.adj.signif. (ns: $p > 0.05$, *: $p < 0.05$, **: $p < 0.01$, ***: $p < 0.001$, ****: $p < 0.0001$)

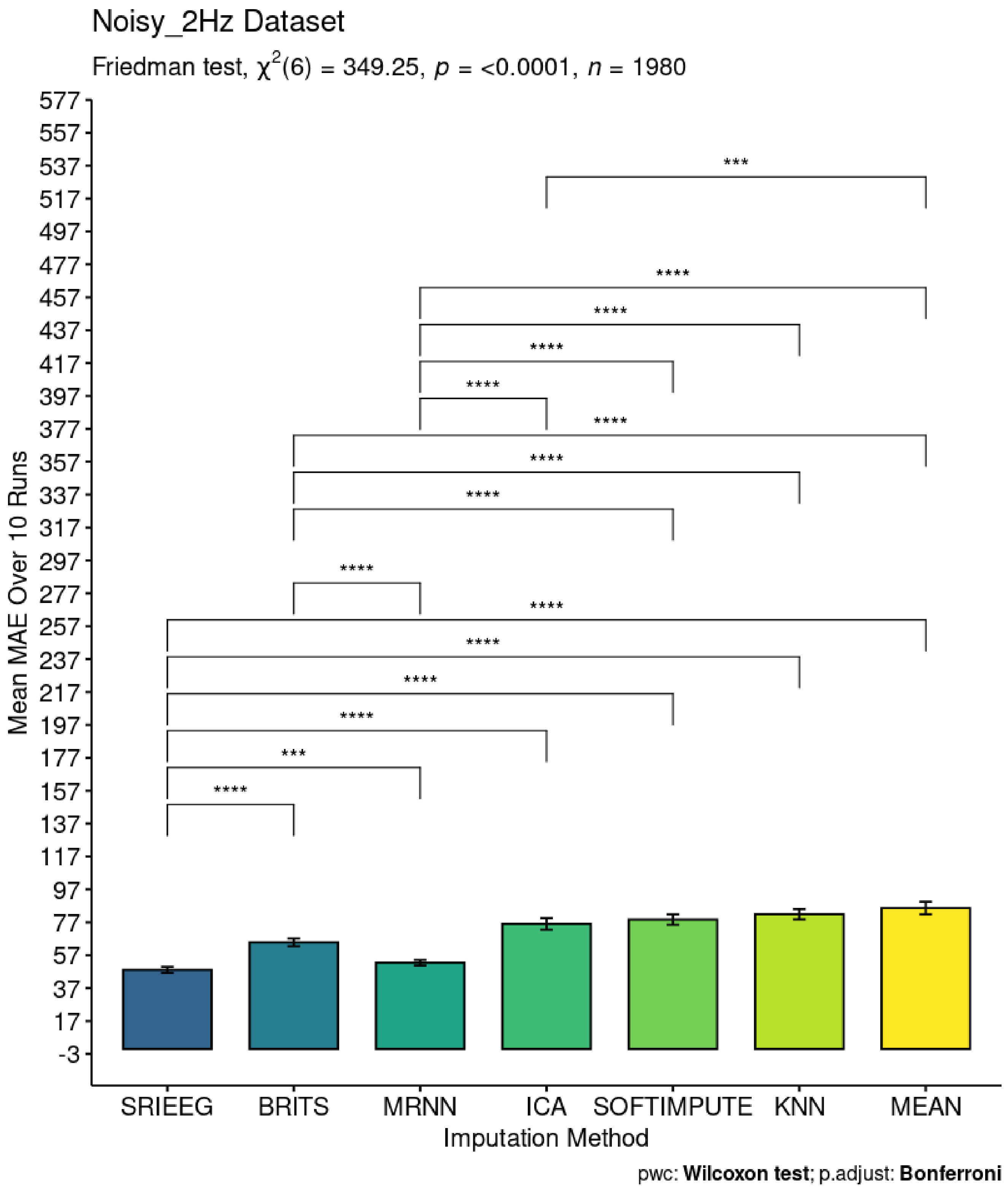

Figure S4. Plot of Noisy_2Hz dataset means over 10 repetitions of mean absolute errors over all imputed values for each imputation method. Error bars show 95% confidence intervals. Significance brackets show results of pair-wise Bonferroni-corrected Wilcoxon signed-rank test.

	Group1	Group2	Estimate	Conf.low	Conf.high	p	p.adj	p.adj.signif
1	SRIEEG	MEAN	-4.33	-4.61	-4.06	4.5 e-168	9.45e-167	****
2	SRIEEG	KNN	-4.55	-4.84	-4.25	1.89e-147	3.97e-146	****
3	SRIEEG	SOFTIMPUTE	-10.2	-10.8	-9.73	5.53e-204	1.16e-202	****
4	SRIEEG	ICA	-4.58	-4.81	-4.35	7.55e-180	1.59e-178	****
5	SRIEEG	BRITS	-2.90	-3.12	-2.68	6.27e-117	1.32e-115	****
6	SRIEEG	MRNN	-3.33	-3.59	-3.07	2.33e-120	4.89e-119	****
7	MEAN	KNN	0.0550	-0.340	0.455	7.82e- 1	1 e+ 0	ns
8	MEAN	SOFTIMPUTE	-5.15	-5.80	-4.49	1.35e- 51	2.84e- 50	****
9	MEAN	ICA	0.195	-0.145	0.545	2.59e- 1	1 e+ 0	ns
10	MEAN	BRITS	1.91	1.56	2.27	7.28e- 27	1.53e- 25	****
11	MEAN	MRNN	1.48	1.11	1.85	3.2 e- 15	6.72e- 14	****
12	KNN	SOFTIMPUTE	-5.33	-5.77	-4.89	2.22e-113	4.66e-112	****
13	KNN	ICA	-0.325	-0.565	-0.0651	1.4 e- 2	2.94e- 1	ns
14	KNN	BRITS	1.44	1.15	1.73	1.35e- 22	2.84e- 21	****
15	KNN	MRNN	1.42	1.08	1.77	2.15e- 15	4.51e- 14	****
16	SOFTIMPUTE	ICA	5.33	4.82	5.84	1.18e- 85	2.48e- 84	****
17	SOFTIMPUTE	BRITS	6.78	6.29	7.28	4.61e-145	9.68e-144	****
18	SOFTIMPUTE	MRNN	7.06	6.55	7.58	1.77e-125	3.72e-124	****
19	ICA	BRITS	1.71	1.43	1.98	5.4 e- 32	1.13e- 30	****
20	ICA	MRNN	1.31	0.995	1.63	2.07e- 15	4.35e- 14	****
21	BRITS	MRNN	-0.270	-0.570	0.0250	7.3 e- 2	1 e+ 0	ns

Table S5. Wilcoxon signed-rank test on Kaggle dataset. Performance averaged over 10 runs on the Kaggle test set. Group1 and Group2 columns list the pair-wise compared methods. Comparisons involving SRI-EEG are highlighted in yellow. The Estimate column lists the median over the pair-wise differences between 10-run-average absolute errors at each imputed value using the Group1 and Group2 methods. Conf.low and Conf.high are the confidence interval for the Estimate. P-values are in the column p. The adjusted p-values using Bonferroni correction are shown in the column p.adj. The significance level of adjusted p-values are in the column p.adj.signif. (ns: $p > 0.05$, *: $p < 0.05$, **: $p < 0.01$, ***: $p < 0.001$, ****: $p < 0.0001$)

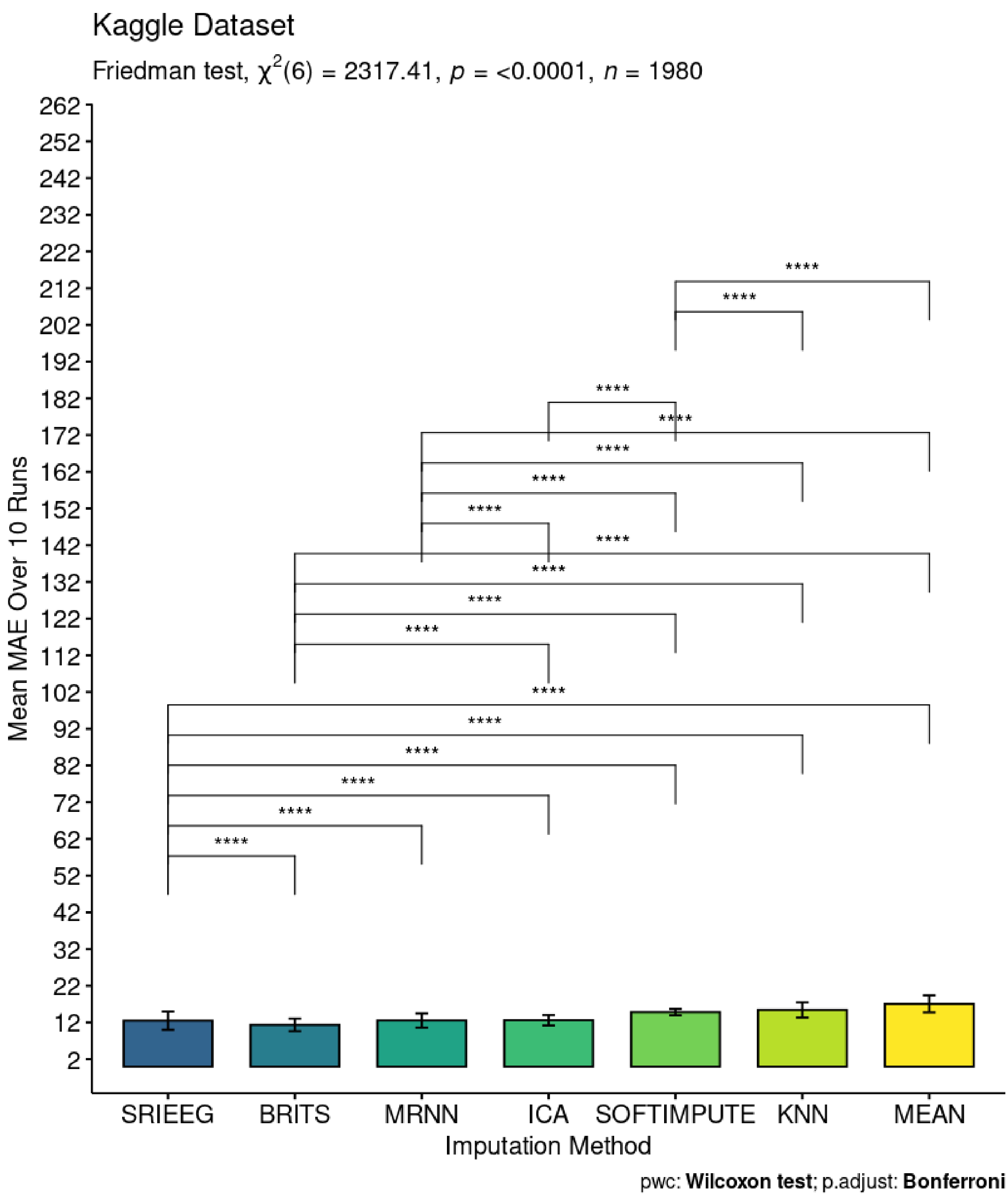

Figure S5. Plot of Kaggle dataset means over 10 repetitions of mean absolute errors over all imputed values for each imputation method. Error bars show 95% confidence intervals. Significance brackets show results of pair-wise Bonferroni-corrected Wilcoxon signed-rank test.

	Group1	Group2	Estimate	Conf.low	Conf.high	p	p.adj	p.adj.signif
1	SRIEEG	MEAN	-1.13	-1.20	-1.07	4.86e-233	1.02e-231	****
2	SRIEEG	KNN	-0.446	-0.487	-0.406	3.8 e-117	7.98e-116	****
3	SRIEEG	SOFTIMPUTE	-0.229	-0.260	-0.198	3.78e- 51	7.94e- 50	****
4	SRIEEG	ICA	-0.164	-0.189	-0.139	6.66e- 38	1.4 e- 36	****
5	SRIEEG	BRITS	-0.168	-0.174	-0.162	0	0	****
6	SRIEEG	MRNN	-0.240	-0.268	-0.212	6.68e- 64	1.4 e- 62	****
7	MEAN	KNN	0.660	0.615	0.705	4.33e-166	9.09e-165	****
8	MEAN	SOFTIMPUTE	0.865	0.800	0.935	2.14e-137	4.49e-136	****
9	MEAN	ICA	0.975	0.905	1.04	7.15e-170	1.5 e-168	****
10	MEAN	BRITS	0.965	0.900	1.03	1.27e-172	2.67e-171	****
11	MEAN	MRNN	0.900	0.830	0.970	8.99e-140	1.89e-138	****
12	KNN	SOFTIMPUTE	0.210	0.165	0.255	4.91e- 21	1.03e- 19	****
13	KNN	ICA	0.295	0.250	0.340	1.16e- 43	2.44e- 42	****
14	KNN	BRITS	0.285	0.245	0.330	3.63e- 43	7.62e- 42	****
15	KNN	MRNN	0.235	0.190	0.280	5.5 e- 25	1.16e- 23	****
16	SOFTIMPUTE	ICA	0.0850	0.0500	0.120	7.07e- 7	1.48e- 5	****
17	SOFTIMPUTE	BRITS	0.0700	0.0400	0.105	2.61e- 5	5.48e- 4	***
18	SOFTIMPUTE	MRNN	-0.00000601	-0.0400	0.0350	9.11e- 1	1 e+ 0	ns
19	ICA	BRITS	0.00493	-0.0250	0.0300	8.27e- 1	1 e+ 0	ns
20	ICA	MRNN	-0.0750	-0.105	-0.0401	3.35e- 6	7.04e- 5	****
21	BRITS	MRNN	-0.0800	-0.115	-0.0500	1.64e- 7	3.44e- 6	****

Table S6. Wilcoxon signed-rank test on SMR dataset. Performance averaged over 10 runs on the SMR test set. Group1 and Group2 columns list the pair-wise compared methods. Comparisons involving SRI-EEG are highlighted in yellow. The Estimate column lists the median over the pair-wise differences between 10-run-average absolute errors at each imputed value using the Group1 and Group2 methods. Conf.low and Conf.high are the confidence interval for the Estimate. P-values are in the column p. The adjusted p-values using Bonferroni correction are shown in the column p.adj. The significance level of adjusted p-values are in the column p.adj.signif. (ns: $p > 0.05$, *: $p < 0.05$, **: $p < 0.01$, ***: $p < 0.001$, ****: $p < 0.0001$)

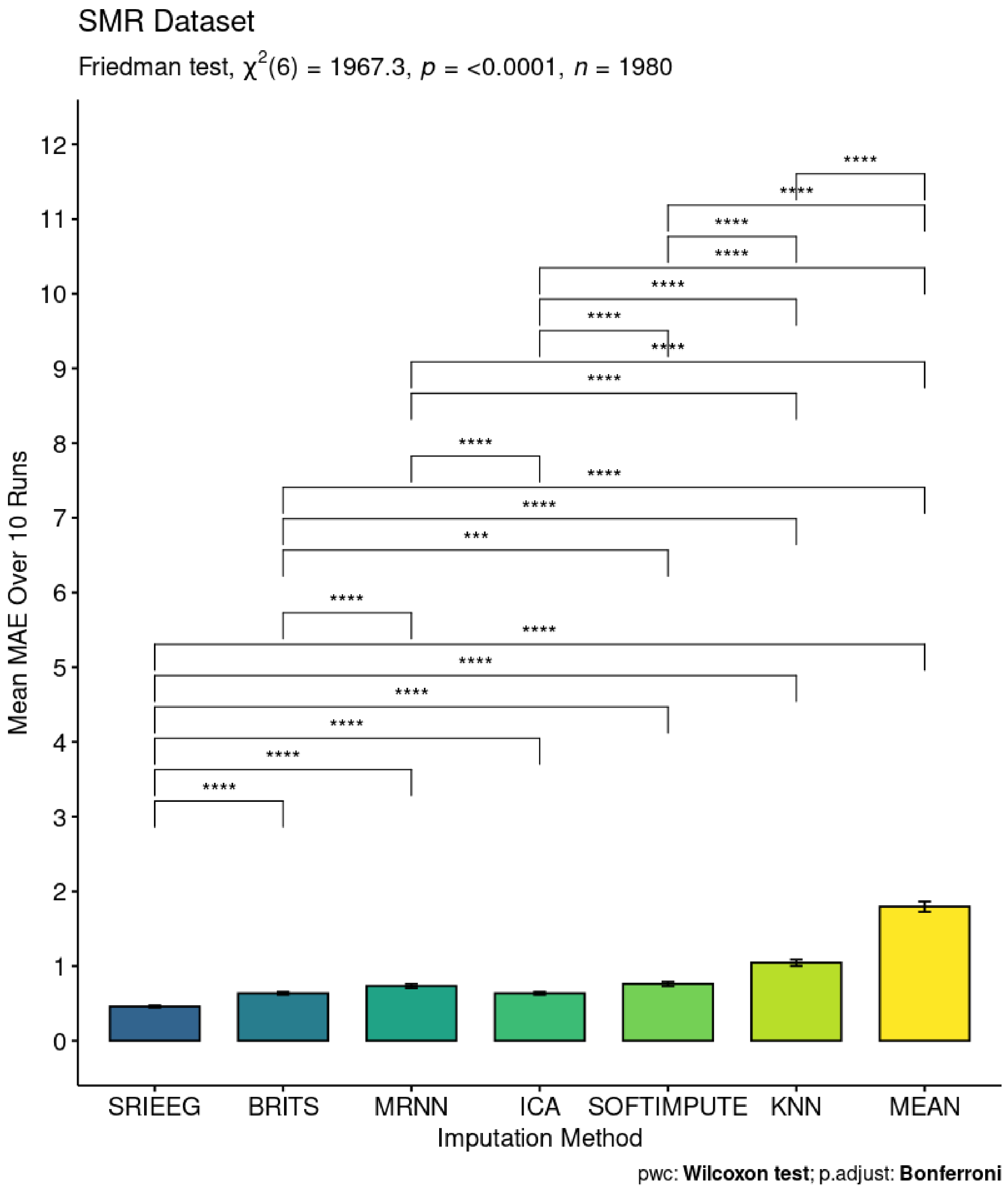

Figure S6. Plot of SMR dataset means over 10 repetitions of mean absolute errors over all imputed values for each imputation method. Error bars show 95% confidence intervals. Significance brackets show results of pair-wise Bonferroni-corrected Wilcoxon signed-rank test.

2 SIGNIFICANCE ANALYSIS: MCNEMAR'S TEST

This section presents the McNemar's test results for Table 8 in the paper. We performed the test on the test set of four datasets: 1) Denoised_1Hz, 2) Denoised_2Hz, 3) Noisy_1Hz, and 4) Noisy_2Hz. The corresponding test results are in Table S7, Table S8, Table S9, and Table S10, respectively. For the test results of each dataset, the table details pair-wise method comparisons.

From our analyses, we see SRI-EEG shows statistical significance over all the compared methods on all the evaluated datasets. This significance can accompany the precision, recall and F-1 score reported in our paper to support SRI-EEG's efficiency on post-imputation analysis, specifically the EEG classification problem.

Method1	Method2	p	Significance
Imputation w/ SRIEEG	No Imputation	8.49e-03	**
Imputation w/ SRIEEG	Imputation w/ BRITS	1.03e-03	**
Imputation w/ SRIEEG	Imputation w/ MRNN	4.03e-02	*
No Imputation	Imputation w/ BRITS	7.18e-01	ns
No Imputation	Imputation w/ MRNN	4.86e-01	ns
Imputation w/ BRITS	Imputation w/ MRNN	2.79e-01	ns

Table S7. McNemar's test on the test set of Denoised_1Hz dataset over 1200 classified labels. Method1 and Method2 are pair-wise compared methods. P-values are in the column p. ns: p > 0.05, *: p < 0.05, **: p < 0.01.

Method1	Method2	p	Significance
Imputation w/ SRIEEG	No Imputation	2.03e-02	*
Imputation w/ SRIEEG	Imputation w/ BRITS	6.50e-03	**
Imputation w/ SRIEEG	Imputation w/ MRNN	4.90e-02	*
No Imputation	Imputation w/ BRITS	8.78e-01	ns
No Imputation	Imputation w/ MRNN	4.61e-01	ns
Imputation w/ BRITS	Imputation w/ MRNN	3.49e-01	ns

Table S8. McNemar's test on the test set of Denoised_2Hz dataset over 1200 classified labels. Method1 and Method2 are pair-wise compared methods. P-values are in the column p. ns: p > 0.05, *: p < 0.05, **: p < 0.01.

Method1	Method2	p	Significance
Imputation w/ SRIEEG	No Imputation	1.33e-03	**
Imputation w/ SRIEEG	Imputation w/ BRITS	1.05e-05	****
Imputation w/ SRIEEG	Imputation w/ MRNN	8.07e-03	**
No Imputation	Imputation w/ BRITS	3.56e-01	ns
No Imputation	Imputation w/ MRNN	4.86e-01	ns
Imputation w/ BRITS	Imputation w/ MRNN	1.09e-01	ns

Table S9. McNemar's test on the test set of Noisy_1Hz dataset over 1200 classified labels. Method1 and Method2 are pair-wise compared methods. P-values are in the column p. ns: p > 0.05, **: p < 0.01, ****: p < 0.0001.

Method1	Method2	p	Significance
Imputation w/ SRIEEG	No Imputation	4.09e-03	**
Imputation w/ SRIEEG	Imputation w/ BRITS	1.85e-03	**
Imputation w/ SRIEEG	Imputation w/ MRNN	3.10e-02	*
No Imputation	Imputation w/ BRITS	9.60e-01	ns
No Imputation	Imputation w/ MRNN	4.15e-01	ns
Imputation w/ BRITS	Imputation w/ MRNN	4.07e-01	ns

Table S10. McNemar's test on the test set of Noisy_2Hz dataset over 1200 classified labels. Method1 and Method2 are pair-wise compared methods. P-values are in the column p. ns: p > 0.05, *: p < 0.05, **: p < 0.01.
